# Path to Actinorhodin: Regio- and Stereoselective Ketone Reduction by a Type II Polyketide Ketoreductase Revealed in Atomistic Detail

**DOI:** 10.1021/jacsau.2c00086

**Published:** 2022-04-07

**Authors:** Stefano A. Serapian, John Crosby, Matthew P. Crump, Marc W. van der Kamp

**Affiliations:** † School of Biochemistry, University of Bristol, University Walk, Bristol BS8 1TD, United Kingdom; ‡ School of Chemistry, University of Bristol, Cantock’s Close, Bristol BS8 1TS, United Kingdom

**Keywords:** polyketide synthesis, protein−protein docking, computational enzymology, 2D-NMR, QM/MM

## Abstract

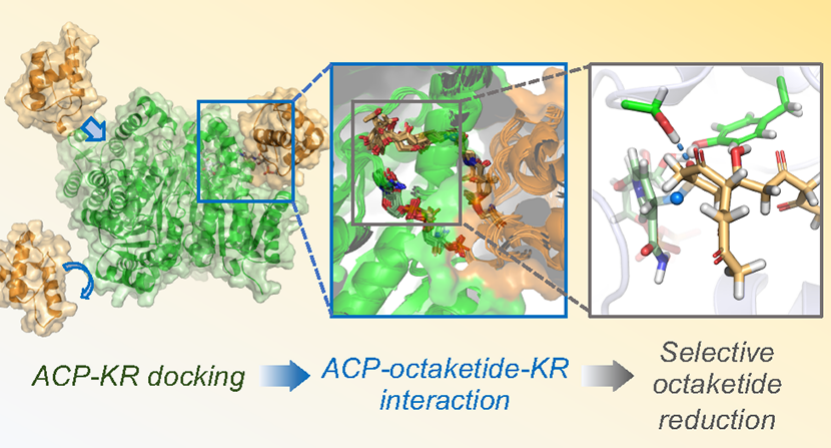

In type II polyketide synthases (PKSs), which typically biosynthesize several antibiotic and antitumor compounds, the substrate is a growing polyketide chain, shuttled between individual PKS enzymes, while covalently tethered to an acyl carrier protein (ACP): this requires the ACP interacting with a series of different enzymes in succession. During biosynthesis of the antibiotic actinorhodin, produced by *Streptomyces coelicolor*, one such key binding event is between an ACP carrying a 16-carbon octaketide chain (*act*ACP) and a ketoreductase (*act*KR). Once the octaketide is bound inside *act*KR, it is likely cyclized between C7 and C12 and regioselective reduction of the ketone at C9 occurs: how these elegant chemical and conformational changes are controlled is not yet known. Here, we perform protein–protein docking, protein NMR, and extensive molecular dynamics simulations to reveal a probable mode of association between *act*ACP and *act*KR; we obtain and analyze a detailed model of the C7–C12-cyclized octaketide within the *act*KR active site; and we confirm this model through multiscale (QM/MM) reaction simulations of the key ketoreduction step. Molecular dynamics simulations show that the most thermodynamically stable cyclized octaketide isomer (7*R*,12*R*) also gives rise to the most reaction competent conformations for ketoreduction. Subsequent reaction simulations show that ketoreduction is stereoselective as well as regioselective, resulting in an *S*-alcohol. Our simulations further indicate several conserved residues that may be involved in selectivity of C7-12 cyclization and C9 ketoreduction. Detailed insights obtained on ACP-based substrate presentation in type II PKSs can help design ACP-ketoreductase systems with altered regio- or stereoselectivity.

## Introduction

**Scheme 1:**
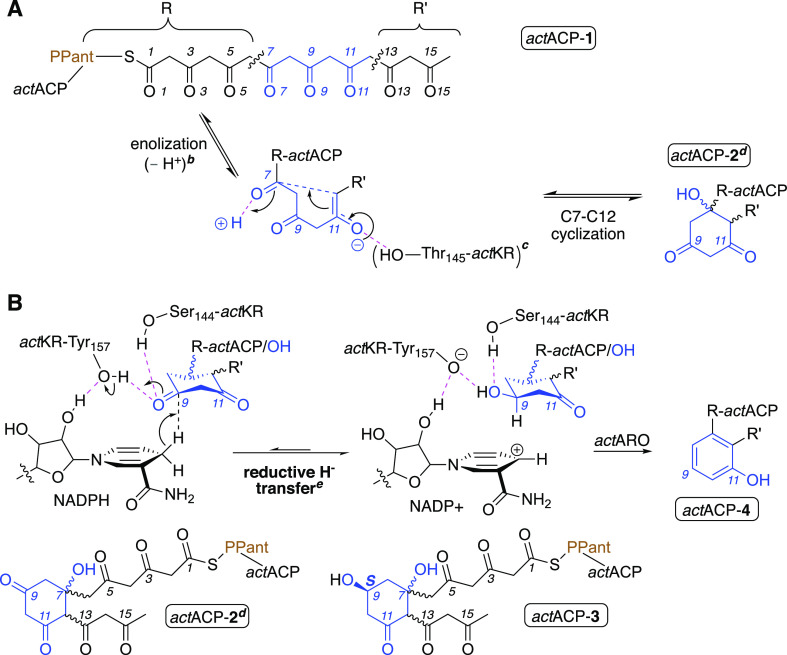
Formation of Cyclized Octaketide 2 (A) and Subsequent Reactions (B) Atoms in blue denote the portion of PPant-octaketide (**1**) forming the six-membered ring in the cyclized intermediate (**2**). Dotted magenta lines denote hydrogen bonds. ^b^Loss of a proton on C12. ^c^The exact role of *act*KR:Thr145 in cyclization is not established. ^d^The Si-face of C9 in *act*ACP-**2** is facing the reader. ^e^Hydride is depicted attacking from the Re-face of C9, giving an *S*-alcohol (i.e., “from below”; defined as “pro-*S* attack”; see chirality assignment in Supporting Information).

**Chart 1:**
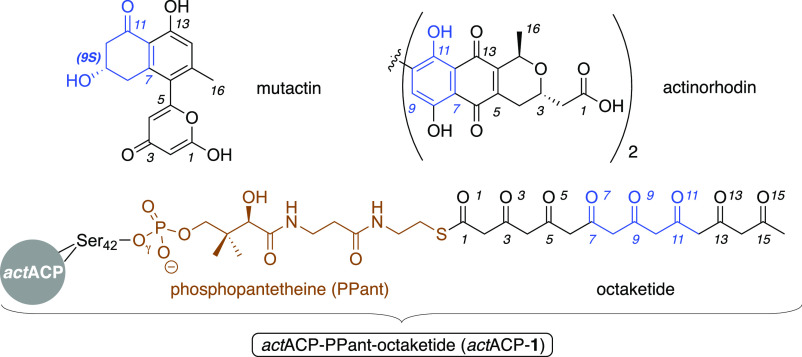
*Act*PKS octaketide and its products.a

In type II polyketide synthases (PKS),^1^ a series of standalone enzymes grow a finite-sized polyketide chain and typically convert it into a complex natural product. An acyl carrier protein (ACP) in conjunction with a chain-length factor (CLF) and ketosynthase (KS) heterodimer catalyze the basic carbon backbone assembly and define a minimal PKS complex.^1,2^ The polyketide alternates between being thioester linked to the KS or the phosphopantetheinyl (PPant) prosthetic group of the ACP (Chart 1) during each round of Claisen condensation of malonyl ACP and the KS bound polyketide. The ACP also shuttles the elongated substrate between subsequent tailoring PKS components that begin the defined series of chemical transformations that include reduction, cyclization, aromatization, dimerization, and numerous other functionalizations.^2,3^ The archetypal type II PKS of the actinomycete bacterium *Streptomyces coelicolor* (*act*PKS)^2−4^ biosynthesizes the antibiotic actinorhodin (Chart 1) from a linear 16-carbon octaketide chain (**1**). After chain elongation, the actinorhodin ketoreductase (*act*KR) likely specifically cyclizes the polyketide between C7 and C12 (**2**) and reduces the ketone at C9 (**3**), while it remains attached to the actinorhodin ACP (*act*ACP) (Scheme 1). Extensive work by Tsai and co-workers has found the KR to be capable of remarkable regio- and stereocontrol^5−8^ and this class of enzymes has attracted interest for their use as biocatalysts in the stereoselective synthesis of small chiral alcohols from achiral ketones.^9−14^ KRs can be mutated to alter and tune their biocatalytic properties as standalone enzymes or used in “combinatorial biosynthesis” alongside other enzyme components from a PKS to produce polyketide derivatives with novel functionality,^15−17^ for example, with altered regio- or stereochemistry.

a Atoms in blue denote the portion of PPant-octaketide (**1**) that forms the six-membered ring upon cyclization.

**Figure 1:**
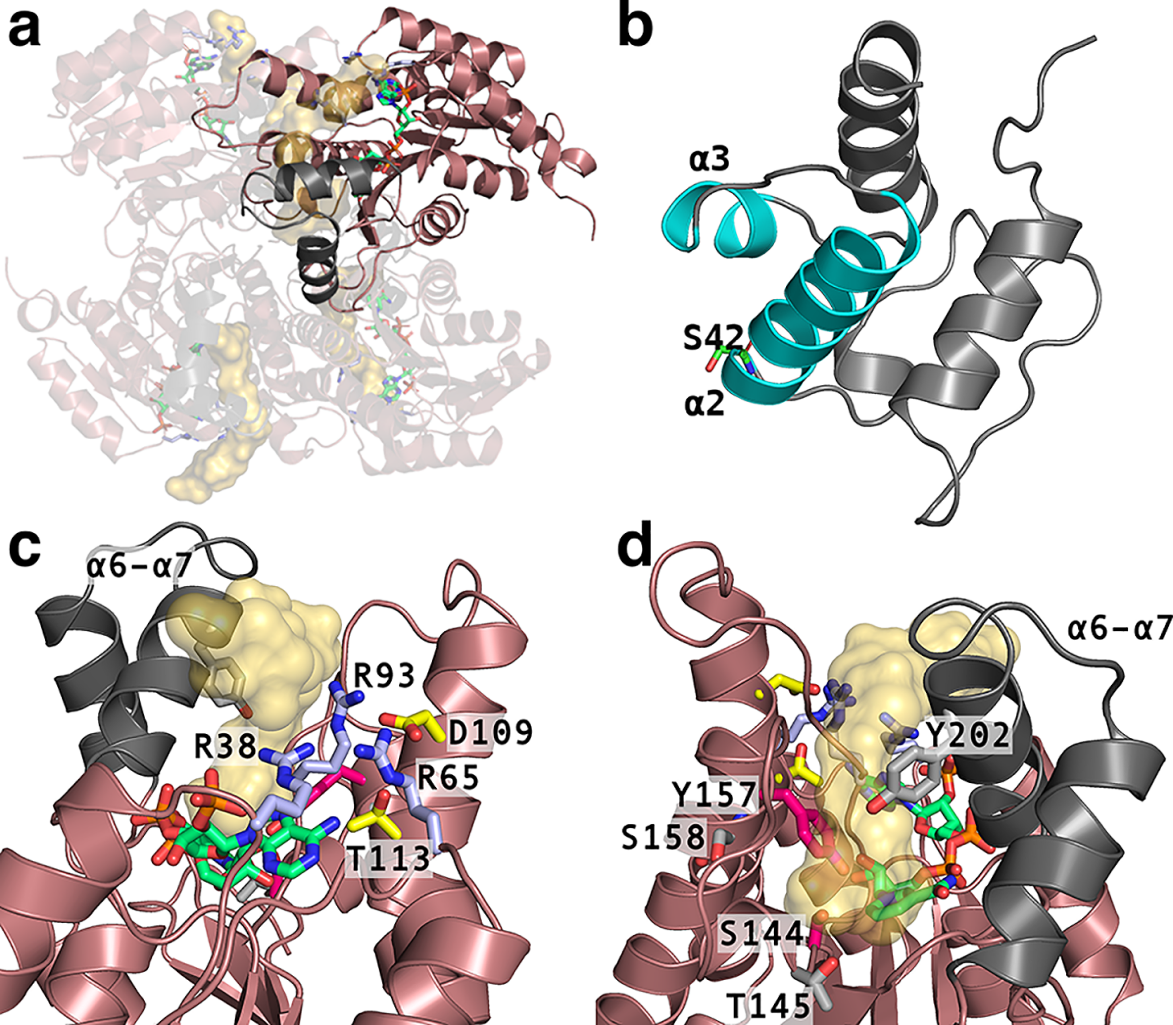
Features of *act*KR (PDB 2RH4)^8^ and *act*ACP (PDB 2MVU)^25^ related to *act*KR-*act*ACP-substrate complex formation. (a) *act*KR tetramer, with NADPH’s C atoms in green, arginine patch (R38, R65, and R93) in light blue, and binding sites for the substrate as transparent orange surfaces highlighted in each monomer. (b) *act*ACP with the α2 and α3 helices in cyan and the PPant-bearing Ser42 highlighted. (c) *act*KR monomer (chain D) viewed from the side of the arginine patch, with D109 and T113 shown (C atoms in yellow). α6−α7 helices and loop are shown in dark gray. (d) As (c) with 180° rotation highlighting putative catalytic residues for cyclization (**1** to **2**; T145, S158, and Y202; C atoms in light gray) and ketoreduction (**2** to **3**; S144, and Y157; C atoms in magenta).

Experimental and theoretical studies have confirmed that the PPant group,^18^ the α2 helix,^19^ and the α3 helix (acting as a conformational “gatekeeper”; Figure 1b)^20^ are all crucial in recognition of PKS and fatty acid synthase (FAS) ACPs^21^ by their enzymatic partners.^22^ In the actinorhodin system, the labile post-assembly octaketide substrate may be partially protected by *act*ACP,^23^ while being transported to the *act*KR.^1^ The *act*ACP shuttle binds to one monomer of the homotetrameric *act*KR, whereupon **1** is unsheathed^1^ into its active site, which is characteristically narrow and restrictive.^5^ In the specific case of the *act*KR–*act*ACP interaction, it is known through mutagenesis studies that recognition is mediated by an “arginine patch” formed by Arg38, Arg65, and Arg93^5,24^ (Figure 1) that binds to the PPant phosphate. The pocket with the arginine patch further contains Asp109 and Thr113 (Figure 1c).^5,8^

The first transformation that likely takes place in *act*KR is the cyclization of *act*ACP**-1** (once spontaneously enolized) to yield *act*ACP-**2** (Scheme 1).^5^ The combination of *act*ACP-**1** binding to *act*KR monomers via the arginine patch^6,8^ and octaketide **1** docking inside the *act*KR’s long but narrow active site^5,6^ probably allows the enzyme to exert strong regiocontrol that favors a C7 and C12 ring closure. C7–C12 cyclization is evident in the final product actinorhodin and the shunt product mutactin formed by the action of only the minimal PKS and *act*KR (Chart 1). In the absence of the *act*KR, C10–C15 cyclization of **1** competes with the natural C7–C12 ring closure.^5,6^ Structure–activity relationships and sequence conservation led to Thr145 and Ser158 being proposed to play a role in this regiocontrol.^5^ Thr145 has been suggested to play a role in stabilizing the O11 enolate, while Ser158 may assist in the proton transfer to O7 from the solvent.

The main (second) transformation in *act*KR—its ketoreduction of *act*ACP-**2**—is both regio- and stereoselective,^5^ producing an alcohol group on C9 (Scheme 1). The first and rate-determining^26^ step in this reaction involves hydride transfer from the *act*KR-bound NADPH to C9^7,27^ and (asynchronous concerted) proton abstraction by O9 from *act*KR/Tyr157. Stabilization is provided throughout the reaction by a hydrogen bond between O9 and *act*KR/Ser144 (Scheme 1). The chirality set by *act*KR at C9 remains unresolved; both **2** and **3** are too labile to be isolated. **3** is shuttled as *act*ACP-**3** to actinorhodin aromatase (*act*ARO) and aromatized to *act*ACP-**4**, leading to a loss of the stereochemical information. In the absence of *act*ARO, mutactin is generated, but its chirality has not been unambiguously confirmed; its designation as “9*S*” in Chart 1 (see chirality assignment in Supporting Information) is based on previous supposition.^6^

It has therefore not yet been possible to infer which stereoisomer and conformer of *act*ACP-**2**, if any, is preferentially formed and reduced within *act*KR; nor how. Therefore, to fully understand the factors that connect the regio- and stereoselectivity, both the protein–protein and protein-substrate interactions between *act*KR and *act*ACP-**1** should be considered in detail. Although binding models have been suggested,^5,7,24^ no crystal structure of the complex exists and detail on the interactions between *act*ACP and *act*KR is lacking. Most solved enzyme-ACP complexes^28−41^ feature FASs,^28−34,41^ and only two feature a PKS component (namely, KS).^35,36^ Moreover, to study protein–substrate interactions, a complex with *act*ACP-**1** is required, but this is unfeasible. In this work, we combine protein–protein docking, molecular dynamics (MD) simulations, 2D protein-NMR spectroscopy, and hybrid quantum mechanics/molecular mechanics (QM/MM) simulations to obtain detailed information on *act*KR–*act*ACP binding and *act*KR–octaketide interactions: our aim is to provide a unified picture of *act*KR structure and function and address the lack of fundamental knowledge on type II PKSs.^1,17,22^

## Materials and Methods

### Protein–Protein Docking

Rigid docking calculations of *act*KR–NADPH and apo *act*ACP were performed using the Bristol University Docking Engine (BUDE),^42^ with GPU acceleration. The structure for *act*KR–NADPH was obtained from previous simulations^27^ starting from PDB ID 2RH4,^8^ and the structure of *act*ACP was taken from model 13 of the NMR ensemble PDB ID 2MVU,^25^ wherein the octaketide mimic is most unsheathed into the solvent (further details in Supporting Information). To maximize docking efficiency, the search space was restricted to areas of each protein’s accessible surface interfaces and excluded areas too far away from the arginine patch. For docking, a “generation zero” of 4600 poses was randomly generated for each of the 43625 possible pairs of chosen *act*KR–NADPH and *act*ACP surface points. The 50 highest-scoring poses were evolved into 2500 “generation-one children” using a Monte Carlo algorithm, and the process (50 new parents, 2500 new children) was repeated to generation five, resulting in ∼43000 fifth-generation binding modes. Seventeen binding modes (labeled **M4–M20**) were selected (based on BUDE score and the distance of *act*ACP/Ser42 to the arginine patch), and for comparison, three *act*KR–*act*ACP models obtained or derived from previous works^5,24,31^ (**M1–M3**) were included. Detailed procedures and coordinates for all models are provided as Supporting Information.

### MD Simulations of *act*KR–*act*ACP Complexes

**Figure 2:**
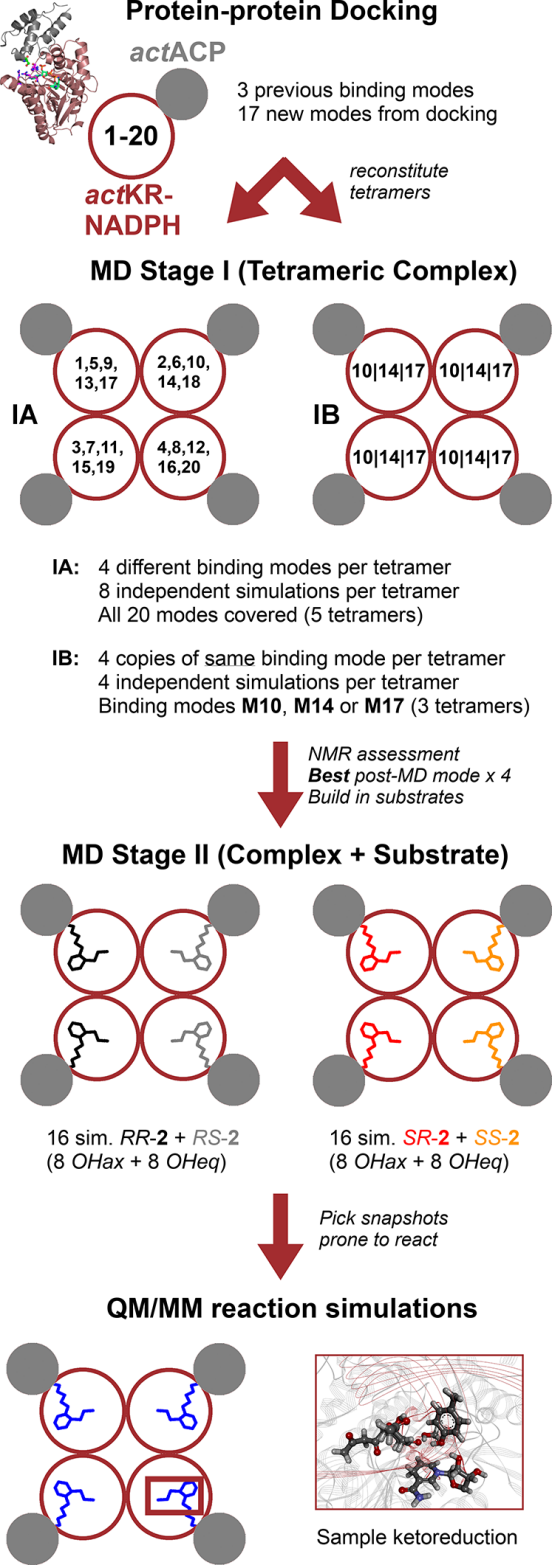
Overview of the computational procedure for *act*ACP-*act*KR model generation and validation. Protein–protein docking is followed by MD simulations in the absence of **2**. After assessment using *act*ACP–*act*KR NMR titration data, MD simulations in the presence of **2** and hybrid QM/MM reaction simulations are performed.

**Scheme 2:**
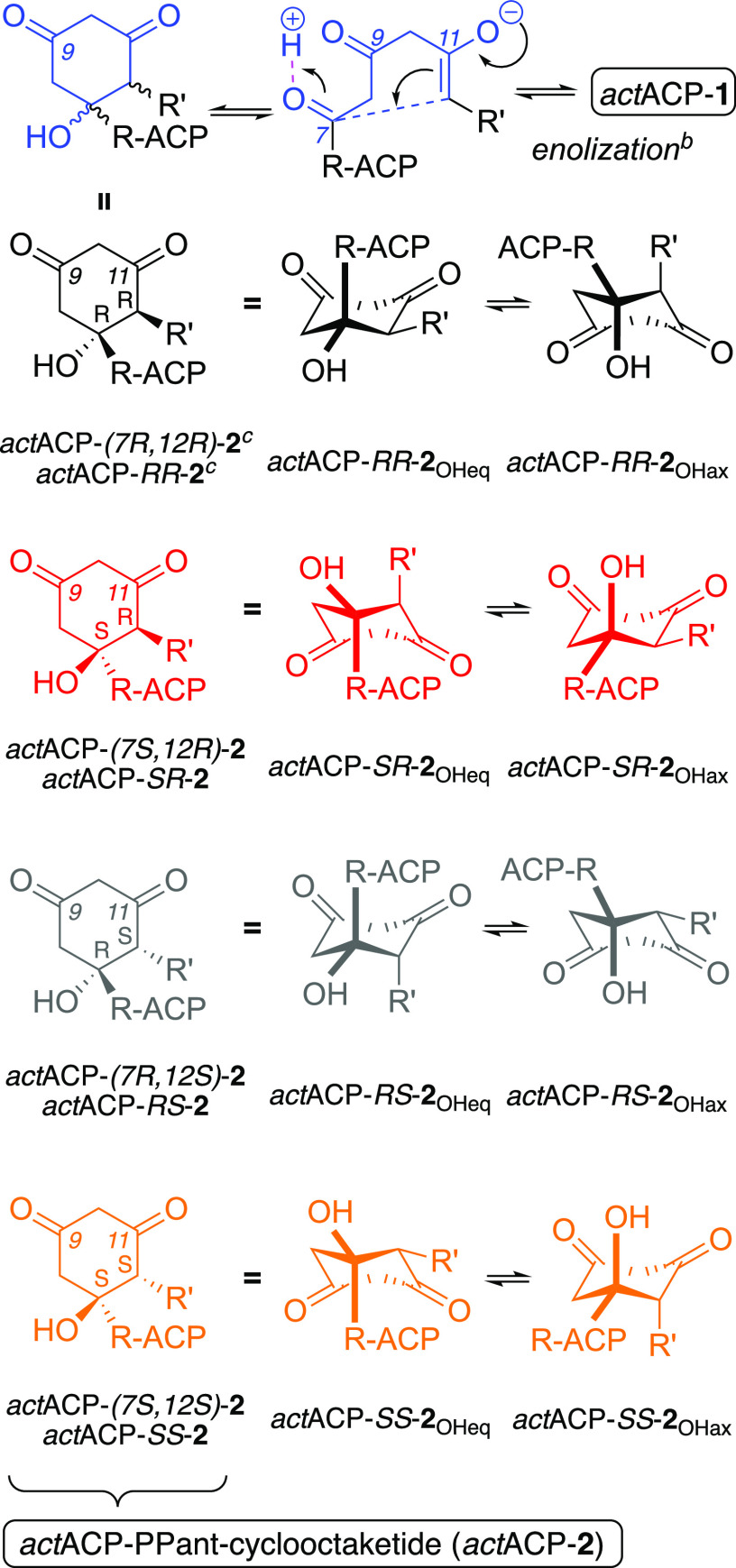
C7–C12 Cyclization of the Natural Substrate of *act*KR with Its Possible Stereoisomers and Chair Conformers Atoms in blue denote the portion of PPant-octaketide (**1**) forming the six-membered ring in the cyclized intermediate (**2**); all structures on the left have the Re-face of C9 facing the viewer (unlike Scheme 1). ^b^Loss of a proton on C12. ^c^Structure of *act*ACP-*RR*-**2** shown in full as Supporting Information, wherein we review its chirality assignment at C7 and C12.

Tetrameric (*act*KR–NADPH)_4_–(*act*ACP)_4_ structures for molecular mechanical (MM) MD simulations were assembled from the docking results, initially using different docking models (**M1–M20**) at each *act*KR chain in the tetramer, without the PPant-octaketide (see Figure 2, series I_A_). For three docking models that gave the most promising results in series I_A_ (**M10**, **M14,** and **M17**), further simulations were run with four *act*ACPs from the same model bound to one *act*KR tetramer (see Figure 2, series I_B_). For the model selected after NMR assessment (**M14**), further MD simulations of the tetrameric complex were performed after introducing the PPant-octaketide moiety (**2**), with all combinations of the possible cyclization conformers (stage II, Figure 2; Scheme 2; Table S3). **2** was modeled from different starting positions in the active sites of each system by finding a balance between: (1) conformational agreement with the PPant of octaketide mimics crystallized with KRs^43−45^ and (2) maintaining catalytic interactions with residues Ser144 and Tyr157. The latter was not possible with C9 positioned for pro-*R* hydride attack (i.e., attack from the Si-face, which would yield an *R*-alcohol at C9); starting structures therefore were modeled for pro-*S* attack in all cases (i.e., attack from the Re-face, resulting in an *S*-alcohol, as depicted in Scheme 1B).^6^ Moreover, while all ketone groups on **2** are potentially prone to keto–enol tautomerization, C=O groups 1, 3, 5, 9, 11, 13, and 15 on all isomer conformers of **2** were always modeled as carbonyls, to keep our work computationally tractable.

Compared to the MD simulations without PPant-octaketide, the α6-α7 loops of actKR and adjacent residues (188–229) were positioned in a more “closed” form, as suggested previously,^5^ with the Tyr202 side chain projecting inside the active site (and a water molecule bridging Tyr202 and the octaketide), as indicated by the recently obtained *act*KR-octaketide mimic structure;^45^ see details in the Supporting Information.

For both stage I and II MD, all residues were in their standard protonation states (consistent with p*K*_a_ predictions from PROPKA 3.1)^46^ with *act*KR His162 protonated on Nδ1 and His153 and His201 on Nε2 (according to the surrounding H-bond network). All systems were solvated in a rectangular box extending at least 11 Å from any protein atom and neutralized by the addition of Na^+^ ions. The *ff14SB* force field^47^ and the TIP3P model^48^ were used, alongside NADPH parameters from Holmberg and co-workers.^49^ GAFF^50^ parameters with HF/6–31(d) RESP point charges were used for the PPant-Ser42 fragment (details and libraries in SI; calculations in ioChem-BD).^51,52^ Multiple independent 32 ns periodic-boundary MD runs were performed in the *NpT* ensemble (after an equilibration procedure), using a 2 fs timestep (with SHAKE for bonds containing hydrogen). The temperature was maintained at 303 K, in line with kinetic assays^8^ and recommended assessment of protein–protein docking stability,^53^ and pressure at 1 atm. All simulations are conducted using AMBER 16^54,55^ with GPU acceleration where applicable. CPPTRAJ^56^ is used for trajectory analysis and post-processing. Further details on generation of starting structures and MD procedures are provided in Supporting Information.

### QM/MM Reaction Simulation of Ketoreduction

QM/MM MD Umbrella Sampling (US) reaction simulations were run with *sander* from AMBER 16.^57,58^ Simulation conditions were identical to the MM MD production runs, except for a shorter time-step (1 vs 2 fs) and no *SHAKE* restraints^59^ on the QM region. This region was limited to one active site and comprised the cyclooctaketide moiety of **2** from C4 to C16; Ser144 and Tyr157 side chains from Cβ; and the nicotinamide moiety of NADPH up to the first ribose (Figure S2). The QM region was treated with the semiempirical method PM6^60^ as used and benchmarked in our previous study on *act*KR (PM6 overestimates the barrier, but the mechanism is correct).^27^ QM/MM MD US simulations of reductive hydride transfer from NADPH to **2**’s C9 were run as previously reported,^27^ using the difference (*x* – *y*) as the reaction coordinate, where *y* is the distance NADPH: H^–^–**2**/C9 and *x* is the distance NADPH: H^–^–NADPH/C_H–_ (Figure S2). Simulations were started from 11 or 12 different “reactive” or “reaction competent” conformations selected from stage II MD runs for each of the three isomers of **2** for which reaction competent conformations were regularly sampled (vide infra). The reaction coordinate was followed using US windows 0.1 Å apart until reaching 1.8 Å, and free energy profiles were obtained by combining all sampling (∼1 ns per isomer) using the weighted histogram analysis method.^61,62^ Further details are reported in the Supporting Information.

### 2D-NMR Titration of *act*KR into *act*ACP

*act*KR^5^ and uniformly ^15^N-labeled *act*ACP^25^ were expressed and purified as described previously. All NMR data were acquired with a Varian INOVA 600 MHz spectrometer at 25 °C. Titrations of *act*KR into ^15^N-labeled holo-*act*ACP were monitored by ^1^H–^15^N HSQC-TROSY experiments. The molar ratios of KR/ACP at each titration point were 0.08, 0.47, 0.33, 0.67, 1.00, 1.34, 1.67, and 2.34, respectively. Stock solution of KR was 1.66 mM KR in 100 mM potassium phosphate pH 6.5, 10 mM EDTA, and 1 mM DTT. This was added to 500 μl of 0.5 mM ^15^N-labeled ACP in the same buffer.

## Results and Discussion

### Approach for *act*KR–*act*ACP Model Generation and Validation

To obtain and validate a reliable, detailed structural model for *act*ACP–**2** binding to the tetrameric *act*KR, a stepwise computational procedure was followed (Figure 2), integrated with NMR spectroscopy. This general approach is in line with recent recommendations^1,63^ on integrative structural biology studies of protein–protein and −substrate interactions, whereby evidence from spectroscopic techniques is typically pieced together with computational techniques (in this case, molecular dynamics and docking). The approach is also in line with a previous work on related systems.^1,64,65^ First, protein–protein docking was used to explore potential *act*KR–*act*ACP binding modes. Selected modes were then refined^53^ through extensive classical molecular dynamics simulations, in the absence of the PPant-substrate (Figure 2; Stage I). Structural analysis based on chemical shift perturbations (CSPs) of *act*ACP obtained from 2D ^1^H–^15^N HSQC *act*KR titration data helped select the most likely binding mode. Then, all four stereoisomers of **2** were modeled into this binding mode, using all eight possible cyclized species, referred to as “isomer-conformers” (see Scheme 2 below). Thereafter, detailed molecular mechanical and hybrid QM/MM molecular dynamics simulations test the enzyme–substrate interactions and expected reactivity (final two stages in Figure 2). In the following subsections, we describe results from each stage in more detail and discuss how the validated model informs on the origins of stereo- and regioselectivity of *act*KR-*act*ACP.

### *Act*KR–*act*ACP Binding Poses from Docking and MD Simulation

Previous work^5,8^ has indicated that the *act*ACP–*act*KR interaction is guided by a patch of three arginines on *act*KR (Figure 1), which recognize and bind the PPant phosphate attached to Ser42 in *act*ACP-**1**.^5,24^ Extensive protein–protein docking of an *act*KR monomer (with NADPH bound) and *act*ACP (in the absence of substrate) was assessed (protein–protein docking in Figure 2) alongside two previously suggested binding modes (**M1**^24^ and **M2**)^5^ and one (**M3**) derived from the crystal structure of *Escherichia coli* enoyl reductase FabI complexed to its ACP (PDB 2FHS).^31^ By considering a combination of the BUDE docking score (i.e., an approximate assessment of the *act*ACP–*act*KR interaction energy in each model, from here on referred to as *BUDE* interaction energy) and a cutoff for the distance [*d*(PPant–*act*KR)] between *act*ACP/Ser42/Cβ (bound to the Oγ, which carries the PPant) and *act*KR/Arg38/Cζ (representing the arginine patch), henceforth referred to as Ser42-patch distance, we selected 17 further docking models (**M4–M20**) that are structurally distinct (see Supporting Information).

The full set of models **M1–M20** were then further assessed using classical molecular dynamics (MD; MD Stage I in Figure 2) simulations to compensate for the rigidity in docking and to help eliminate false positives.^53^ For each model, 8 MD-refined binding poses were obtained through 8 independent MD simulations of 32 ns and subsequent clustering (series **I**_**A**_ in Figure 2, details in Supporting Information). For each MD refined binding pose (8 for each of the 20 docking models, i.e., 160 in total), BUDE scores and *d*(PPant–*act*KR) were measured (Figure S4). We then use (arbitrary) BUDE interaction energy and *d*(PPant–*act*KR) thresholds of <−90 kJ mol^–1^ and <9 Å, respectively, to select for poses that (1) are likely to occur with reasonable frequency and (2) are in line with PPant-octaketide insertion into the KR channel. Based on this, binding modes **M1–M3**, **M5–8**, **M11**, **M12,** and **M19** were deemed unlikely to be representative after MD refinement: all poses from **M2** and **M3** both had large Ser42-patch distances and unfavorable BUDE interaction energies; all poses from **M1** and **M5–8** had Ser42-patch distances >9 Å; all poses from **M11**, **M12,** and **M19** had consistently poor BUDE interaction energies (>−90 kJ mol^–1^).

**Figure 3:**
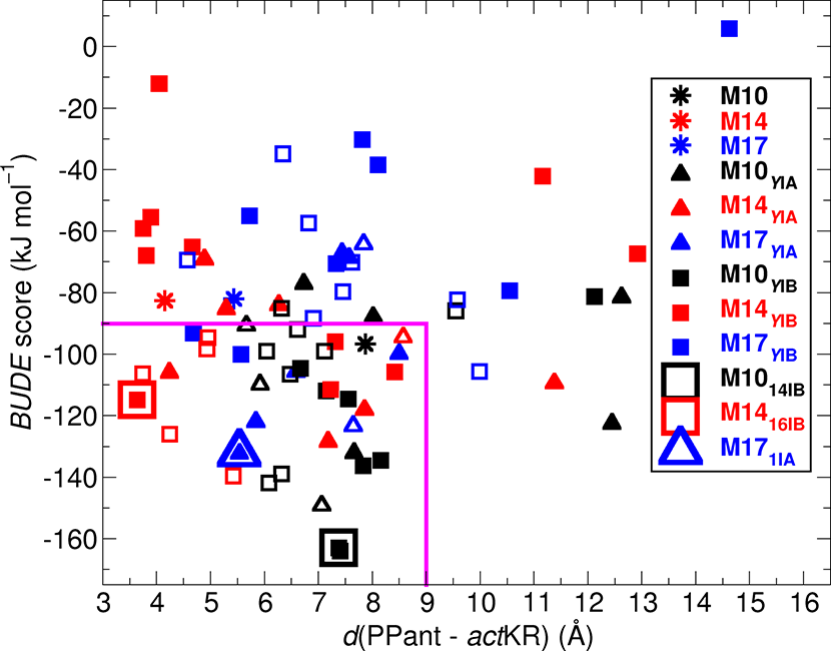
Refinement and ranking of docking models by MD simulation. *Act*KR–*act*ACP binding modes originating from MD simulations of M10 (black); M14 (red); and M17 (blue) are shown with their interaction energy (BUDE score, *y*-axis) and Ser42-patch distance [*d*(PPant–*act*KR), *x*-axis]. Asterisks denote original docking modes; squares, those originating from series IA (M*X*_YIA_; *X* = 10, 14, 17; *Y* = 1–8); and triangles, those originating from IB (M*X*_YIB_; *Y* = 1–16). The area bound by magenta lines indicates the region with BUDE score < −90 kJ mol^–1^ and *d*(PPant–*act*KR) < 9 Å thresholds. Open triangles and squares refer to binding modes whose Ser42-patch distance deviates by more than 15% from its average value in the last 4 ns of the MD simulations from which they originate. The three binding modes selected for structural analysis and validation with NMR are highlighted by framed symbols.

MD refinement of the remaining docking models (**M4**, **M9**, **M10**, **M13–M18,** and **M20**) yielded several examples of binding poses with greater thermodynamic likelihood and compatibility with PPant insertion [i.e., low BUDE interaction energy and Ser42-patch distance, *d*(PPant–*act*KR)]. When applying thresholds of BUDE interaction energy below −90 kJ mol^–1^ and Ser42-patch distance below 9 Å (pink rectangle in Figure 3), there was one likely binding pose each originating from docking models **M4**, **M9**, **M15**, **M16**, and **M20** (Figure S4); two originating from **M13** (Figure S4); three from **M18** (Figure S4); and as many as four from **M10** and **M14** and five from **M17** (Figure S4; triangles in Figure 3). All but two of these 23 refined poses improved their BUDE scores from docking, indicating that the flexibility introduced by MD simulation led to a more plausible *act*KR–*act*ACP binding interface.

The only models with >50% of MD-refined snapshots within the thresholds, and therefore more likely to occur than others, were docking models **M10**, **M14,** and **M17**. These were selected for further MD simulation (series **I**_**B**_ in Figure 2; note that the next-best model **M18** is structurally similar to **M14**, with a RMSD between *act*ACP/Cα atoms of only 1.59 Å, and was therefore not selected). Simulations were performed using only one of each binding mode at each *act*KR–*act*ACP interface (using 4 × 32 ns simulations for each tetramer, Figure 2). Clustering then gave 16 additional representative snapshots for each binding mode. Using the same thresholds as before [BUDE score < −90 kJ mol^–1^; *d*(PPant–*act*KR) < 9 Å], 13 additional binding poses were found for **M10**; 9 for **M14**, and just two for **M17** (Figures 3 and S5). (All but one of the additional poses again improved their BUDE interaction energy from docking.) Notably, even for these three poses that frequently exhibit favorable BUDE interaction energies, much less favorable interaction energies (≫−90 kJ mol^–1^; Figures 3 and S4 and S5) also occur within 32 ns of MD simulation. This likely reflects a transient *act*KR–*act*ACP binding interaction.

**Figure 4:**
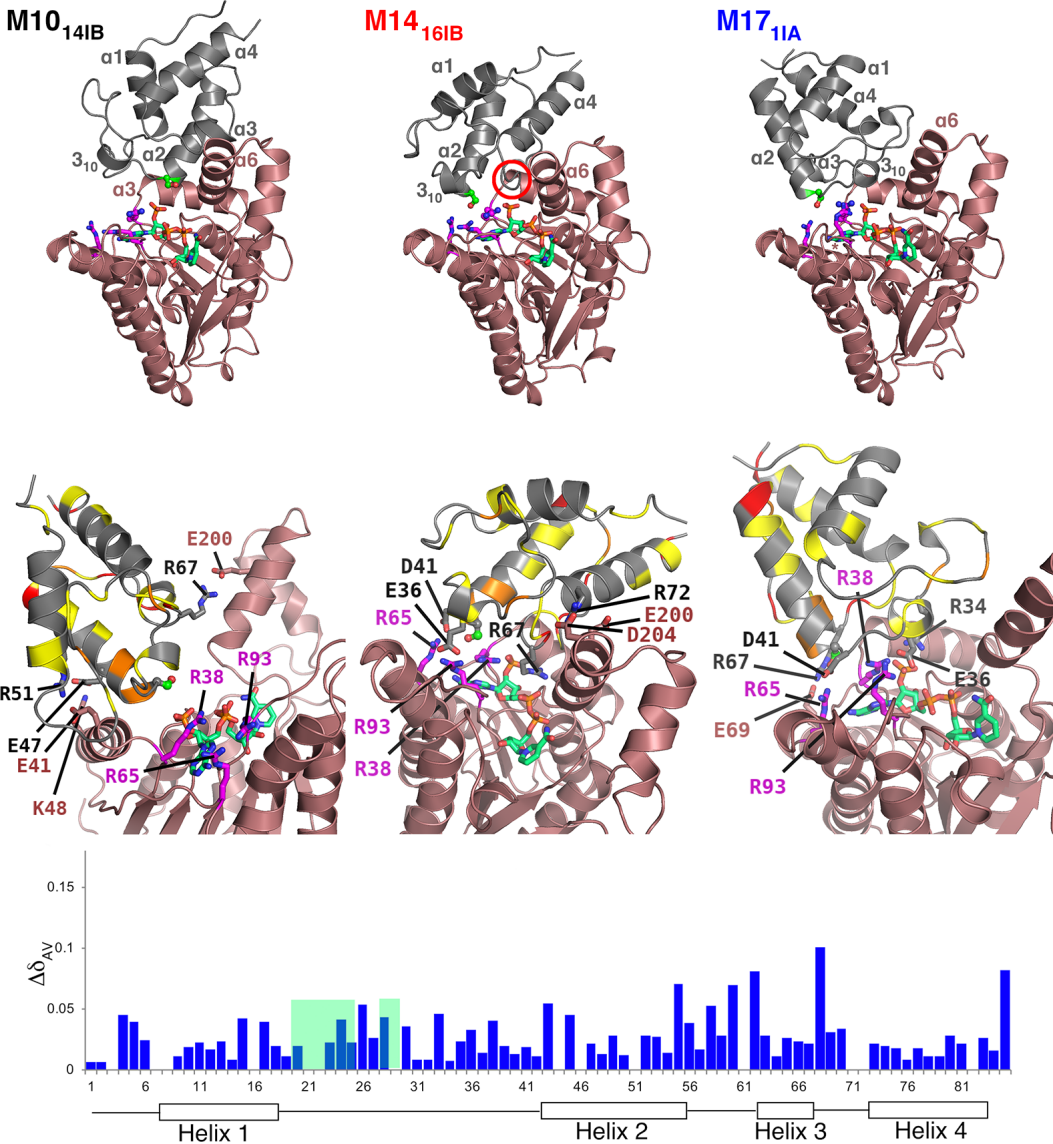
Comparison of putative *act*ACP–*act*KR binding modes **M10**_**14IB**_, **M14**_**16IB**_, and **M17**_**1IA**_ and NMR titration data ^15^N-labeled *act*ACP with *act*KR. Top: overview of *act*ACP–*act*KR binding modes, with *act*KR (off-red cartoon) in the same orientation; *act*ACP as gray cartoon. In **M14**_**16IB**_, *act*ACP’s “gatekeeper” α3 helix is marked by a red circle; it is central to the *act*ACP–*act*KR interface and has lost some of its structure. NADPH, *act*ACP/Ser42, and the *act*KR arginine patch and *act*ACP residues implicated in salt bridges (Table S5) are labeled and rendered as sticks: NADPH with C atoms in green; Arginine patch with C atoms in magenta; *act*ACP/Ser42 in ball-and-stick with bright green C atoms; and H atoms omitted for clarity. Middle: magnification of the *act*ACP–*act*KR interfaces with the ACP backbone colored according to the magnitude of the measured CSPs [as change (Δ) in weighted averages δ_AV_] upon addition of *act*KR (from KR/ACP ratio of 0.08 to 2.34): 0.02 < Δδ_AV_ < 0.04 ppm in yellow, 0.04 < Δδ_AV_ < 0.06 ppm in orange, and Δδ_AV_ ≥ 0.06 ppm in red. Bottom: Δδ_AV_ values for every *act*ACP residue. δ_AV_ is given by (δ_AV_ = {0.5[Δδ(^1^H)^2^ + (0.2Δδ(^15^N))^2^]}^1/2^);^66^ where Δδ_AV_ values are missing, this indicates either no significant shift, residues without −NH (Pro61, Pro71) or that assignments for these residues were tenuous. Full NMR data (^1^H–^15^N HSQC) are shown in Figure S6.

To further narrow down the selection of binding poses to those that are consistent with PPant-octaketide insertion, we monitored whether the Ser42-patch distance remained within 15% of its original value during the last 4 ns of the MD simulation from which each pose was selected (Figure 3; filled-in symbols). Combining this criterion with the most negative BUDE score and the shortest Ser42-patch distance resulted in the selection of refined poses **M10**_**14IB**_, **M14**_**16IB**_, and **M17**_**1IA**_ (see framed symbols in Figure 3 and structures in Figure 4). The subscripts denote the MD replica (number 14, 16, or 1) and series (I_A_ or I_B_). This selection should ensure that the three selected models are thermodynamically likely (favorable BUDE interaction energy; stability until the end of their MD runs) representations of possible (transient) *act*ACP–*act*KR interaction modes, which are in agreement with PPant phosphate recognition by the arginine patch.^5,24^

### NMR and Structural Analyses of the *act*ACP–*act*KR Interaction

All three thermodynamically plausible *act*ACP–*act*KR binding modes selected after docking and MD simulation (Figure 4) feature *act*ACP/Ser42 (at the N-terminus of *act*ACP’s α2 helix) relatively close to Arg38. Only in **M14**_**16IB**_ and **M17**_**1IA**_, however, are all three arginines in the patch^5,8^ positioned to capture the phosphate in the PPant moiety of **2** (Figure 4): *act*ACP/Ser42:Oγ is 3.4 and 5.2 Å away from the center of mass of the arginine guanidinium moieties, respectively (vs 12.9 Å away in M10_14IB_). All three binding modes exhibit several electrostatic interactions between *act*ACP and *act*KR (Table S5). In **M10**_**14IB**_, however, none of these interactions are formed with the arginine patch^5,24^ or NADPH (with most contacts between the *act*ACP α2 and *act*KR α6 helices). In contrast, in **M14**_**16IB**_ and **M17**_**1IA**_, charge–charge interactions are formed with the arginine patch by both *act*ACP/Asp41 and *act*ACP/Glu36 and with the phosphate moieties of NADPH by Arg67 (**M14**_**16IB**_) or Arg34 (**M17**_**1IA**_). In **M14**_**16IB**_, *act*ACP α3 is in the center of the *act*ACP–*act*KR interface, whereas the overall binding interaction in **M17**_**1IA**_ is dominated by the α1−α2 loop and does not involve α3.

To compare the plausibility of the binding modes, we conducted ^1^H–^15^N HSQC titration experiments using ^15^N-labeled *act*ACP and unlabelled *act*KR (Figure 4, bottom panel; Figure S6). Titration to an excess of *act*KR/*act*ACP showed small, but distinct CSPs particularly across the α2−α3 loop and α3, consistent with relatively weak binding. The largest magnitude CSPs are observed in this region (I60, D62, and V68) and may also report on conformational changes in α3 as reported previously,^20^ again pointing to the involvement of α3 in the *act*ACP–*act*KR interaction, as observed in **M14**_**16IB**_. Furthermore, residues of the flexible α1−α2 loop from T21-D29 exhibited exchange broadening; this loop is fully solvent-exposed only in **M14**_**16IB**_. Although the interface predominantly characterized by charge–charge interactions (see above) suggests a highly specific molecular recognition, the broadly distributed CSPs overall indicate that the *act*KR/*act*ACP likely forms a weak transient complex in solution. It is therefore likely that many transient *act*KR/*act*ACP binding modes will occur, as opposed to one well-defined protein–protein interface. We note that for the *E. coli* FAS ACP-acyltransferase interface, such structural plasticity has been suggested to be a key contributor to catalytic efficiency.^41^

In summary, structural analysis and NMR titration suggests that binding mode **M14**_**16IB**_ is a good representation of a thermodynamically feasible, transient *act*ACP–*act*KR complex, with the following features: (1) the *act*ACP “gatekeeper” helix (α3) is central to the interface, occupying a cleft above the central NADPH phosphates and adjacent to the arginine patch;^5,24^ (2) the α4 helix interacts with the (mobile) α6 helix of *act*KR; (3) part of the α2−α3 loop, indicated by Hadfield et al. as being important for protein–protein interactions,^24^ is also in contact with *act*KR; and (4) the α1−α2 loop is solvent exposed.

### Reactivity of ACP-Bound Cyclized Octaketides in *act*KR

To assess the possible binding interactions of cyclized octaketides in *act*KR, we performed multiple independent MD simulations of *act*KR–*act*ACP with all possible cyclized conformers of the all-ketone tautomeric form of **2** (MD stage II in Figure 2): C7–C12 cyclization of **1** can, in principle, lead to four different stereoisomers of **2** (color-coded in Scheme 2; see chirality assignment for one isomer in the Supporting Information): (7*R*,12*R*)-**2** (henceforth *RR*-**2**; black); (7*R*,12*R*)-**2** (*RS*-**2**; gray); (7*R*,12*R*)-**2** (*SR*-**2**; red); and (7*R*,12*R*)-**2** (*SS*-**2**; orange). In turn, each of these four isomers can access two low-energy chair conformers (Scheme 2), with the C7–OH substituent oriented either axially (**2**_OHax_) or equatorially (**2**_OHeq_). To include a plausible *act*ACP–*act*KR binding interaction (which will constrain the mobility of the PPant-octaketide), consistent with our NMR titration study, *act*ACP binding mode **M14**_**16IB**_ was used. We note that other binding modes that similarly constrain the PPant-octaketide mobility (such as **M10**_**14IB**_ or **M17**_**1IA**_) would likely lead to similar results. The *act*KR α6−α7 loop was remodeled prior to MD simulation, based on an *act*KR-octaketide mimic complex structure,^45^ in line with the suggested role of this loop in substrate recognition.^5^ By using previous structural information^43−45^ and satisfying contacts between **2** and catalytic residues, initial placements for the PPant and the octaketide moieties were generated (two alternative starting positions were used in order to explore a greater portion of conformational space; modeling details and coordinates are included in Supporting Information).

**Figure 5:**
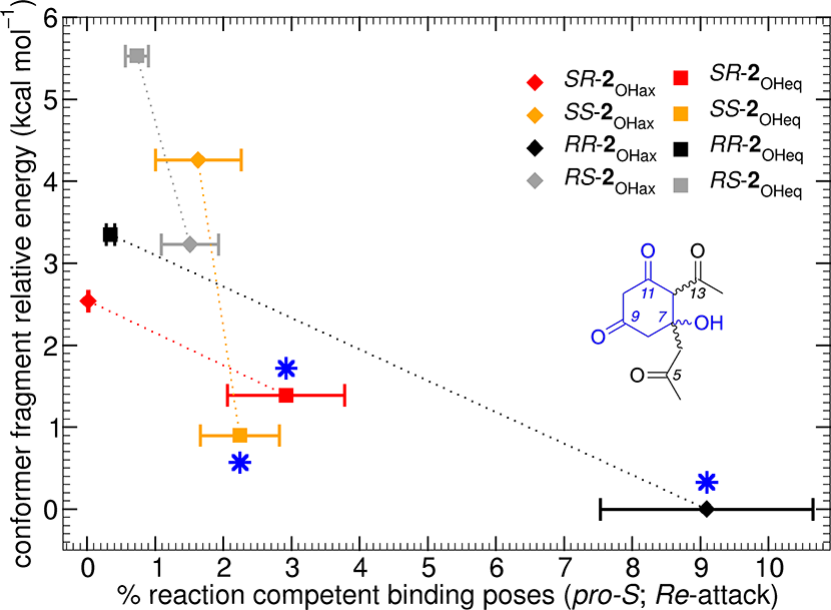
Comparison of QM energies and in-enzyme reaction competent poses of the different isomer conformers of the cyclized octaketide **2**. *X*-axis: percentage of *pro*-*S* reaction competent binding poses (%_reac_) present in stage II MD simulations (see Supporting Information for definition). *Y*-axis: relative free energies of the C4–C14 fragment [SCS-MP2/6-31+G(d,p)//B3LYP/6-31+G(d,p), see Supporting Information for details] of the hydrogen-capped cyclopentaketide fragments (see inset). Lines are shown to guide the eye. (*) marks isomer conformers chosen for QM/MM reaction simulations. Species are color-coded and labeled as in Scheme 2. Error bars along the *x*-axis are based on a leave-one-out procedure (see Supporting Information).

In the resulting MD simulations, the frequency of reaction competent poses (%_reac_, defined by satisfying key distances; see Supporting Information)^67^ of **2** toward C9 ketoreduction was monitored, and the combined %_reac_ values (from 4 active sites × 8 replicas × 32 ns × four initial systems, see Table S3) were compared for each of the eight possible cyclization isomer conformers of **2** (Figure 5 and Table S6). *RR*-**2**_OHax_ had the highest frequency of reaction competent poses (at 9.1%), followed by *SR*-**2**_OHeq_ and *SS*-**2**_OHeq_ (with 2.9% and 2.2%, respectively). The remaining five isomer conformers had less than 2% such poses. Essentially all reaction competent poses are *pro*-*S*; only 12 *pro*-*R* poses were observed for all isomer-conformers (all for *RS*-**2**_OHeq_) out of a total of >1.5 million snapshots. Our simulations thus show that **2** is (much) more prone to *pro*-*S* hydride attack at C9 (from the Re-face, resulting in *S* chirality), in agreement with previous in silico models of the presentation of the polyketide substrate to NADPH^6^ (see further below).

**Figure 6:**
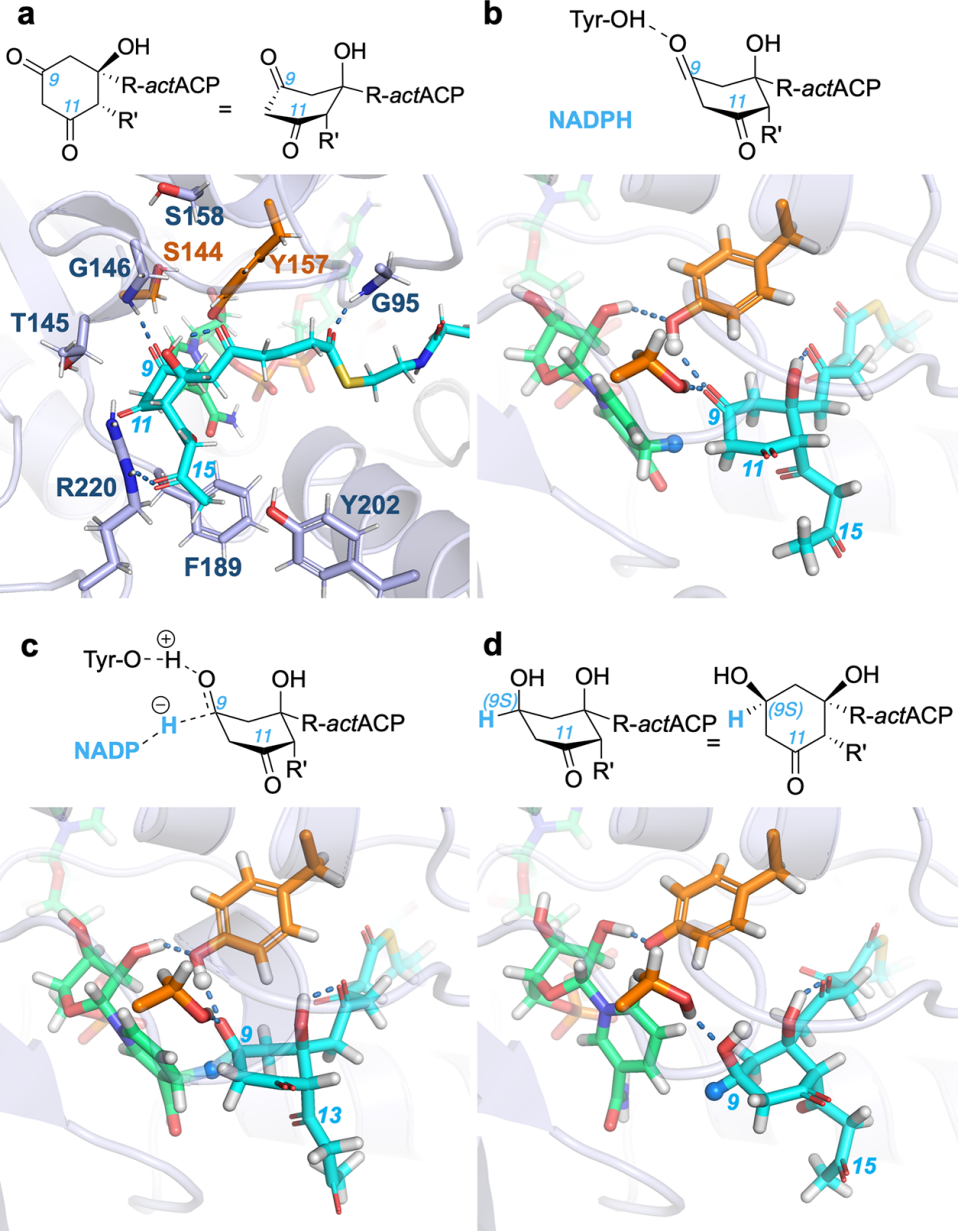
Key steps in the ketoreduction of *act*ACP-*RR*-**2**_OHax_ by *act*KR. The sequence depicts the *pro-S* hydride attack on isomer-conformer *RR*-**2**_OHax_ (i.e., “below”, from the Re-face of C9), with salient octaketide carbons labeled where possible (a) representative “nonreactive” snapshot of *act*ACP-*RR*-**2**_OHax_ (C atoms in cyan) inside the active site of *act*KR, highlighting residues (sticks; C atoms light blue) that could be important for regioselectivity per our hydrogen bond analysis (see text). Catalytic residues Ser144 and Tyr157 (C atoms in orange) and the NADPH cofactor (C atoms in green) are shown. Gly95/NH, part of the XGG motif,^5,6,8^ interacts frequently with **2**/O1; other residues are discussed in the text. (b) Reaction competent pose of *act*ACP-*RR*-**2**_OHax_ poised for hydride transfer from NADPH. Hydride is shown as the blue sphere and *act*KR/Tyr157/Hη (i.e., −(O)H) as the white sphere. (c) Transition state of the ketoreduction reaction, with hydride being transferred from NADPH to **2**/C9. (d) Product of ketoreduction, with Tyr157’s phenolic proton transferred to **2**/O9. Every panel comprises a 2D representation of the C7–C12 ring mimicking its 3D rendering as closely as possible; panels (a,d) also contain representations of the C7–C12 ring with the Si-face of C9 facing the reader. Groups R and R′ are defined in Scheme 1A.

When considering the relative stability (free energy) of all eight isomer conformers (Figure 5, *y*-axis; QM calculation details in Supporting Information, optimized structures in ioChem-BD),^51,52^ we find that there is some degree of correlation between thermodynamic stability (after cyclization) and the propensity to form reaction competent poses in the *act*KR active site for the ensuing ketoreduction step (e.g., Figure 6b): *RR*-**2**_OHax_ is most stable, followed by *SS*-**2**_OHeq_ and *SR*-**2**_OHeq_ (0.9 kcal mol^–1^ and 1.4 kcal mol^–1^ higher in energy, respectively). The remaining isomers are significantly higher in energy (2.5 to 5.6 kcal mol^–1^). The correlation between these chemically distinct quantities was unexpected. Similarly, there is also a correlation between the frequency of reaction competent poses for reduction and thermodynamic stability of cyclization products for the axial versus equatorial C7–OH arrangement (especially for 12*R* isomers): in 7*R* isomers, axial conformers are more stable and attain more reaction competent poses; in 7*S* isomers, the opposite is true. *A priori*, there is no reason why thermodynamic stability of the isomer conformers should correlate with their proneness to react in the successive ketoreduction step.

To simulate the chemical reaction itself, we selected three isomer conformers of **2** (*RR*-**2**_OHax_, *SS*-**2**_OHeq_, and *SR*-**2**_OHeq_). As indicated by the relatively infrequent occurrence of reaction competent poses (at most 9.1% for *RR*-**2**_OHax_), the cyclized octaketide spends the majority of the time “in standby”, that is, bound in the active site with C9 close to the catalytic residues, but not quite ready for reaction (Figure 6a). Moving to a reaction competent conformation (e.g., Figure 6b) will thus come at a slight free energy cost (1.4, 2.1, or 2.3 kcal mol^–1^ at room temperature for *RR*-**2**_OHax_, *SS*-**2**_OHeq_, and *SR*-**2**_OHeq_, respectively, based on Δ*G* = *RT* ln[% reaction competent poses]). For each, we performed combined quantum mechanical/molecular mechanical (QM/MM) MD simulations of ketoreduction at C9 (see QM/MM reaction simulations in Figure 2), using the same approach as our previous work on the reduction of *trans*-1-decalone by *act*KR.^27^ The transition states and reaction barriers obtained here are similar (Figures 6 and S3), which demonstrates that the selected complexes modeled based on **M14**_**16IB**_ can indeed represent reaction competent *act*ACP-*act*KR poses, further validating this MD-refined model. As expected, the transition state corresponds to the hydride transfer between NADPH and C9, concerted with proton transfer from Tyr157 to O9 (Figure 6c). Subsequently, Tyr157 moves to coordinate to a ribose hydroxyl of NADP^+^ (Figure 6d), ready for reprotonation through a proton shuttle likely involving the ribose and Lys161.^26,27^ Notably, our simulations show energetically feasible reactions, while the cyclized octaketide is bound to *act*ACP, confirming that the ACP-PPant tether does not need to be broken prior to ketoreduction by *act*KR (in contrast to what is expected for hedamycin KR).^9^ The barriers to reaction are not significantly different between the three isomer conformers, suggesting that *act*KR can facilitate ketoreduction to a similar extent in all three, via axial hydride attack at C9 (Scheme 1 and Figure 6b-d). While a preference for axial attack is in line with previous findings on the reduction of small alicyclic ketones by agents such as [AlH_4_]^−^ and [BH_4_]^−^,^68,69^ it is in contrast with findings by Østergaard et al. on reduction of the small alicyclic *trans*-1-decalone by another ketoreductase^70^ and our own findings for its reduction by *act*KR itself.^27^ It appears that the tendency of *act*KR to catalyze equatorial H^–^ attack in small, nonendogenous substrates can be overridden by factors such as binding site architecture, spatial constraints arising from *act*KR–*act*ACP binding, and the presence of oxygen substituents on C7 and C11.

### Determinants of actKR Stereo- and Regioselectivity

The overwhelming prevalence of *pro-S* reaction competent poses in our MD simulations (stage II; H^–^ attack from the Re-face) indicates that S-selectivity for ketoreduction at C9 in *act*KR is defined by its active site structure in combination with the position of the incoming PPant chain, which is determined by the *act*KR–*act*ACP interaction, as suggested previously^5,6^ (Figures 1d; 6; S2). The side chains of the adjacent residues *act*KR/Thr145 (possibly stabilizing O11 during cyclization of **1** to **2**; Scheme 2)^5^ and *act*KR/Ser144 (stabilizing O9 during ketoreduction; Scheme 1) form a relatively rigid template close to the nicotinamide ring of NADPH. When O11 and O9 bind to these residues upon arrival of **1** into the active site, C7–C12 cyclization to any isomer conformer of **2** creates spatial constraints that strongly favor reductive hydride attack in a *pro-S* pose (i.e., from the Re-face or “from below” in Figure 6b-d to yield an *S*-alcohol at C9).

We noted above that the link between C7–C12 ring conformer stability and greater propensity for (*pro-S*) C9 ketoreduction is unexpected, indicating that the *act*KR active site might have evolved to preferentially perform reduction on the most stable cyclization isomer conformers *RR*-**2**_OHax_, *SS*-**2**_OHeq_, and *SR*-**2**_OHeq_, that is, those that are more likely to form upon cyclization of **1**. In addition, *act*ARO—likely having evolved in tandem with *act*KR—might prefer the combination of *S* chirality at C9 alongside the three isomer conformers to perform its conversion of **3** to **4** (although confirming this hypothesis would require detailed mechanistic studies of *act*ARO, which is beyond the scope of this work).

Apart from its stereoselectivity in ketoreduction, the other remarkable characteristic of *act*KR is its regioselectivity, namely, why cyclization occurs between C7 and C12 (if it occurs on *act*KR, rather than on *act*KS/CLF) and why ketoreduction then occurs specifically at C9 (with the link between the two already noted).^5^ To investigate if and how the binding site architecture might drive regioselectivity, we examined the formation of hydrogen bonds (direct or water-mediated) between *act*KR and substrate oxygen atoms in MD trajectories from stage II (Figure 6a, full details in Tables S7 and S8). Hydrogen bonds between the cyclized octaketide moiety and *act*KR are rather transient during our simulations. Short-lived hydrogen bonds are consistent with **1** and **3** “sliding” in and out of the binding channel, respectively, as suggested by Javidpour et al.,^5^ as well as the “in standby” conformation of the cyclized octaketide (with catalytically competent poses only being attained for a fraction of the simulation time, Figure 5). Hydrogen bonds directly relevant for ketoreduction at C9 are observed between O9 and Ser144 and Tyr157 on *act*KR (Scheme 1), but not as the most frequent (average frequencies, respectively, of 5.3 and 4.8% for *RR*-**2**, 4.3 and 4.3% for *SR*-**2**, and 3.0 and 1.8% for *SS*-**2**). Instead, the most frequent hydrogen bonding for O9 occurs with nearby backbone hydrogens of *act*KR/Phe189 (a residue whose importance was also noted experimentally)^5,8^ and *act*KR/Gly146 (Figure 6a and Table S7). Interactions with *act*KR/Ser144 and *act*KR/Tyr157’s −OH hydrogens are typically mediated by water bridges when found (Table S8). These interactions are consistent with isomers of **2** being held “in standby” in the binding site (Figure 6a), with the C9=O9 carbonyl never far from reaching a reaction competent pose (Figure 6b). Only this carbonyl interacts with the key catalytic residues, thus achieving regioselectivity at C9.

The simulated isomers of **2** can be considered as the products of C7–C12 cyclization of the all-ketone tautomeric form of **1** (Scheme 2), and their interactions may therefore reflect how such regioselective cyclization might be promoted by the *act*KR active site. One possible key interaction could be hydrogen bonding between O11 and *act*KR/Thr145’s hydroxyl group; however, our simulations only indicate sporadic and indirect hydrogen bond interactions (through water bridges, Table S8). A different hydrogen bond interaction that may be relevant for cyclization, between **2**/O7/H7 and *act*KR/Tyr202’s −OH group, is observed occasionally in simulations for most isomer-conformer pairs (Tables S7 and S8). This (highly conserved)^5^ Tyr202 side chain, in its orientation toward the active site^45^ (Figures 6a and S2), could thus be involved in catalyzing regioselective C7–C12 cyclization, for example, as a proton donor to O7, or aiding proton transfer from the nearby His153 and His201. Other interactions that may be relevant for cyclization are the long-lived intramolecular hydrogen bond between **2**/H7 and **2**/O5, and the occasional water bridges between **2**/O5 and *act*KR/Tyr202’s −OH, both of which may contribute to C7–C12 cyclization through stabilization of proton transfer to O7. Notably, interactions of **2**/O7/H7 with *act*KR/Ser158’s hydroxyl group, previously proposed to play a role in proton donation in cyclization,^5^ are hardly ever sampled. While these specific hydrogen bond interactions detected for O5, O7, and O11 may be structurally and/or electronically important factors for regioselective cyclization of **1** to **2**, further work is required to confirm the possible roles of *act*KR residues in C7–C12 cyclization, such as stabilization of the enolate species and the source for O7 protonation (e.g., involving Tyr202, His153, and/or His201).

Finally, we consider contacts at the extremities of the (cyclized) octaketide species in its all-ketone form. Zhao et al. recently used extensive MD simulations of *act*KR and a double mutant, which affects chain-length specificity, together with octaketide and tetraketide substrate mimics.^45^ They considered two previously proposed substrate entrance sites, a “back-patch” near Q149/R220 and a “front-patch”, identical to the “arginine patch”. In our work, only binding at the latter is considered, as this is enforced by the location of ACP, with the PPant phosphate group binding to the arginine patch. This is consistent with the preference of the PPant octaketide mimic found by Zhao et al.^45^ For the octaketide, we find frequent and fairly persistent hydrogen bonds (direct or through bridging waters) between the start of the chain (O1) and the backbone *act*KR/Gly95/NH (Figure 6a). This glycine is part of the highly conserved XGG motif characterizing type II PKS, which has been suggested to be an anchor point for the PPant-octaketide to be presented to the *act*KR active site.^5,6^ Our simulations further support this. At the other end, O15 forms frequent hydrogen bonds (direct or through bridging waters) with *act*KR/Arg220, located toward the C-terminus of the α7 loop and previously considered by mutagenesis^5^ (Figure 6a). Arg220 can “seal” the binding pocket at its far end (including through hydrogen bonding with *act*KR/Gln149,^8^ forming the “back-patch” that can support binding of short polyketides)^45^ and could thus be a key factor for the regioselectivity of cyclization by helping the linear octaketide **1** buckle upon itself near O15, folding the C12–C16 fragment back onto C7–C11.

## Conclusions

In the type II actinorhodin polyketide synthase, association between actinorhodin ketoreductase (*act*KR) and an actinorhodin ACP (*act*ACP) carrying a phosphopantetheinylated octaketide results in the latter being inserted into the *act*KR active site (as **1** or cyclized as **2**). Subsequently, **2** is stereoselectively reduced at C9=O9 to yield alicyclic chiral alcohol **3**. In this work, we study the *act*ACP–*act*KR binding interaction in atomic detail and suggest a plausible representative binding mode, using a combination of protein–protein docking, molecular dynamics simulations, and NMR CSPs. Then, further molecular dynamics simulations (including QM/MM reaction simulations) based on this binding mode are used to investigate the mechanism and the sources of regio- and stereoselectivity of *act*KR toward its natural substrate.

After initial selection of simulation-refined docking models based on estimated binding affinity and proximity of *act*ACP/Ser42 to a “patch” of three arginines on *act*KR (Arg38, Arg65, and Arg93), one binding mode was found to be most consistent with our 2D NMR data and previous reports. In this mode, *act*ACP docks onto *act*KR with its α3 helix and the N-termini of α-helices 2 and 4. Subsequent simulations based on this binding mode of complexes with all possible C7–C12 cyclization isomers of **2** revealed an overwhelming preference for *pro-S* ketoreduction at C9=O9, particularly for the most thermodynamically stable cyclization isomer [i.e., (7*R*,12*R*)-**2** with C7–OH oriented axially]. In addition to establishing a link between chirality at C7/C12 and chirality at C9, this finding unequivocally confirms previous experimental data on mutactin, inferring that C9 should be enantiopure;^6^ it also strongly suggests that chirality at **3**:C9 should be *S* rather than *R* (i.e., with hydride attack occurring from the C9’s Re-face rather than Si), and that *act*KR preferentially catalyzes this attack axially rather than equatorially. The (transient) binding mode of *act*ACP in conjunction with spatial features of the *act*KR active site are sufficient to cause the indicated *S*-selectivity. QM/MM MD reaction simulations of C9 ketoreduction were performed for the three isomers of **2** that most frequently formed reaction competent binding poses. This indicated that *S*-selective ketoreduction is equally efficient for these isomers (i.e., no specific C7/C12 chirality is preferred in the chemical step) and our model yields energy barriers similar to those obtained with efficiently converted small molecules (further validating our proposed *act*ACP–*act*KR binding mode). Further analysis of our MD simulations of **2** inside *act*KR identified residues (such as Gly95 and Arg220) that are important for steering the binding of the substrate and holding it “in standby” in the *act*KR active site, as well as those that may aid regioselective cyclization between C7 and C12.

In summary, we have combined protein–protein docking, extensive MD simulation, NMR, and QM/MM reaction simulations to produce and validate a detailed model of the *act*KR–*act*ACP interaction that is consistent with all currently available experimental data for cyclization and ketoreduction of the natural octaketide substrate.^5,6^ The model obtained provides important mechanistic insights, demonstrating the use of multiscale atomistic simulations to improve our understanding of biocatalytic protein–protein complexes. We have shown that the specificity of the *act*KR–*act*ACP interaction, together with the architecture of the *act*KR active site, has direct implications for the elegant regio- and stereoselectivity of *act*KR toward its natural substrate. The information obtained can aid in future engineering of type II PKS ketoreductase/acyl carrier systems, for example, to make them process alternative substrates or change cyclization, regio-, and stereoselectivity; an important step toward building biocatalytic systems that can yield new polyketide derivatives with different chain lengths, stereochemistry, and/or cyclization patterns.

## Supporting Information Available

Chirality assignment, additional details on docking calculations, construction of starting structures, parametrization of isomer conformers of **2**, MD simulation and analysis, NMR data, details of QM/MM simulations, and analysis of hydrogen bonds in MD runs in **II** (PDF)
Input files for protein–protein docking; structures of **M1–M20**, **M10**_**14IB**_, **M14**_**16IB**_, and **M17**_**1IA**_ in PDB format; AMBER input (topologies and coordinates) for all runs in **I**_**A**_, **I**_**B**_, and **II**; and modified force field parameters for the PPant moiety and octaketide portions of **2** (ZIP)
