# Supplementary material for: Path to Actinorhodin: Regio- and Stereoselective Ketone Reduction by a Type II Polyketide Ketoreductase Revealed in Atomistic Detail
Source: JACS Au. 2022 Apr 7;2(4):972–84. doi: 10.1021/jacsau.2c00086 (PMC9088766; doi:10.1021/jacsau.2c00086)
Supplement: Supplementary file 1 — au2c00086_si_001.pdf [file au2c00086_si_001.pdf]

SUPPORTING INFORMATION

The Path to Actinorhodin: Regio- and Stereoselective Ketone Reduction by a Type II Polyketide Ketoreductase Revealed in Atomistic Detail

Stefano A. Serapian,[1] John Crosby,[2] Matthew P. Crump[2] and Marc W. van der Kamp[1]*

[1]School of Biochemistry, University of Bristol, University Walk, Bristol, BS8 1TD, United Kingdom

[2]School of Chemistry, University of Bristol, Cantock's Close, Bristol, BS8 1TS, United Kingdom

Corresponding Author

* E-mail: Marc.VanderKamp@bristol.ac.uk

1. Chirality Assignment for Mutactin, one Isomer of **2**, and **3**

Chart S1. Chirality Assignment for Salient Compounds Mentioned in the Main Text.

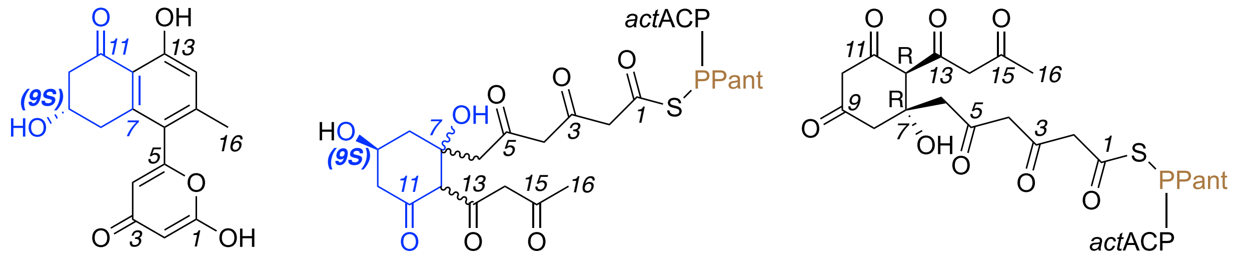

mutactin | *act*ACP-*(9S)*-**3** | *act*ACP-*(7R,12R)*-**2**

For clarity, we provide here the chirality assignment for mutactin (to show *S*-chirality at C9; *cf.* main text, Chart 1), *act*ACP-**3** (to show *S*-chirality at C9; *cf.* Scheme 1B), and the *(7R, 12R)* isomer of *act*ACP-**2** (*RR*-**2**, Scheme 2): all of these are illustrated in Chart S1, with similar representations to those in respective schemes/charts in the main text (Chart 1, Scheme 1B, Scheme 2). Note that the *Re*-face of C9 in *RR*-**2** is facing the viewer.

For mutactin (C9):

- O(H)9 has priority 1 (higher atomic number than C8, C10, H);
- H has priority 4 (lower atomic number compared to C8, C10, O(H)9);
- C8 and C10 are equivalent (both are CH_2 bound to another C);
- Moving to C7 and C11, the latter has priority over the former, *per* Cahn-Ingold-Prelog rules:
  - C11 is considered to be bound to two O (2 × O11) and one C (C12);
  - C7 is considered to be bound to three C (2 × C12, C6).
- As a result, C10 has priority 2 and C8 has priority 3.
- In Chart S1 (and Chart 1), H is in the foreground, and O(H)9 – C10 – C8 are arranged clockwise: were H in the background, they would be arranged counterclockwise, making chirality at C9 *S*.

For *act*ACP-**3** (C9):

- O(H)9 retains priority 1, as in mutactin;
- H retains priority 4, as in mutactin;
- C8 and C10 remain equivalent;
- C11 retains priority over C7, because:
  - C11 is again considered to be bound to two O (2 × O11) and one C (C12);
  - C7 is now bound to one O (O(H)7), and two C (C6, C12).
- As a result, C10 and C8 retain priority 2 and 3, respectively.
- In Chart S1 (and Scheme 1B), H is in the background, and O(H)9 – C10 – C8 are arranged counterclockwise, again making chirality at C9 *S*.

For *act*ACP-*RR*-**2** (C7):

- O(H)7 has priority 1 (higher atomic number than C6, C8, C12).
- C12 has priority 2, because:
  - C6 and C8 are both bound to 1 C and two H; whereas
  - C12 is bound to two C (C11, C13) and one H.
- Moving on from C6 and C8:
  - C5 and C9 are equivalent (both are carbonyls bound to another C);
  - C4 and C10 are equivalent (both are CH_2 bound to another C);
  - C3 and C11 are equivalent (both are carbonyls bound to another C);
  - C2 is superseded by C12, because:
    - C12 is bound to two C (C7, C13) and one H;
    - C2 is bound to one C (C1) and two H.
  - Therefore:
    - C8 has priority 3 and C6 has priority 4.
- Note that in Chart S1 (and in Scheme 2), the highest-priority group (O(H)7) is in the background; chirality at C7 is thus *R*.

For *act*ACP-*RR*-**2** (C12):

- H has priority 4 (lower atomic number than C11, C7, C13).
- C7 has priority 3, because:
  - C7 is bound to one O (O(H)7) and two C (C8, C6); whereas
  - C11 and C13 are both carbonyls bound to another C, and *per* Cahn-Ingold-Prelog rules are therefore considered to be bound to two O and one C.
- Moving on from C11 and C13:
  - C10 and C14 are equivalent (both are CH_2 bound to another C);
  - C9 and C15 are equivalent (both are carbonyls bound to another C);
  - C8 takes priority over C16, because:
    - C8 is bound to two H and one C.
    - C16 is bound to three H.
- C11 therefore has priority 1; and C13 has priority 2.
- In Chart S1 (and Scheme 2), H is in the background, and C11 – C13 – C7 are arranged clockwise, making chirality at C12 *R*.

2. Additional Details on Protein-Protein Docking Calculations

2.1 Starting structures

To account for initial relaxation in an (intracellular) aqueous environment, the reference structure for *act*KR–NADPH was derived from monomer A of a snapshot of classical MD simulations of the *act*KR tetramer ([*act*KR–NADPH]$_4$) with the *trans*-decalone substrate deleted;[1] these in turn were run starting from the *act*KR crystal structure obtained by Korman et al. (PDB 2RH4).[2] The reference for *act*ACP was model 13 from the NMR solution structure ensemble of the ACP bearing a solvent exposed 3,7-dioxo-octyl mimic of **1** (PDB 2MVU).[3] Ser17 was mutated back to the wild-type cysteine, the mimic of **1** was deleted, and the Ser42-OH side chain restored.

2.2 Choice of Surface Points

To avoid generation of unrealistic poses, search space for docking was restricted to areas of the protein surfaces most likely to be involved in interface formation, as indicated by previous work.[2, 4]

Out of 2755 total Connolly surface points[5] generated for *act*ACP by BUDE's *b_gensrf* utility,[6] we selected a subset of 125 points no farther than 8.50 Å from any of the atoms in Ser42.

Similarly, out of 9173 total surface points generated for the isolated holo-*act*KR monomer (*act*KR–NADPH), we chose a subset of 349 points that were: (1) no farther than 7.50 Å from any of the atoms in its Arg-Asp patch;[2, 4] and, simultaneously (2) farther than 3.00 Å from any atom that would be occluded by tetramerisation. The latter was defined as residues in monomers A, B, C, and D whose atoms come closer than 5.50 Å from each other in the reference MD-derived structure; these included, in each monomer, residues 67, 97-104, 107, 108, 111, 115, 116, 119, 120, 123, 126, 127, 132, 148-156, 158-160, 162-167, 169-171, 173, 174, 176, 177, 218-225, 227, 228, 230, 231, 234, 235, 238, and 242-261.

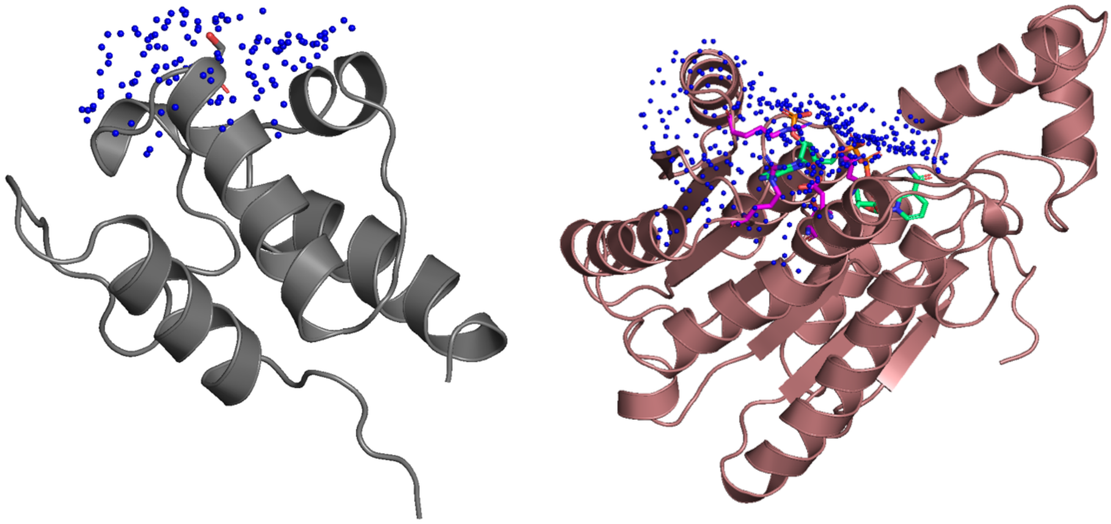

Figure S1. Surface points (in blue) chosen for protein-protein docking, on the *act*ACP (left), and *act*KR–NADPH monomer A (right). For reference, atoms in *act*ACP:Ser42 (C atoms in gray), NADPH (C atoms in green), and the arginine patch and its supporters (*act*KR:Arg38, *act*KR:Arg65, *act*KR:Arg93, *act*KR:Asp109, *act*KR:Thr113; C atoms in magenta) are rendered as sticks: these residues were used to guide our choice of surface points. Key for remaining atoms: O: red, N: blue, P: orange.

Co-ordinates of the chosen points are available as Supporting Information and are illustrated in Figure S1. The choice of 349 surface points on *act*KR–NADPH and 125 on *act*ACP leads to 43625 possible combinations of points as referred to in the main text.

2.3 Results and Ranking Criteria to obtain **M4-M20**

The 17 (*act*KR–NADPH)–*act*ACP binding modes originating from our rigid protein-protein docking (**M4-M20**) were chosen from the 43625 best-scoring fifth-generation "children" generated for each of the possible pairs of chosen *act*KR–NADPH and *act*ACP surface points (Figure S1). In order to select the most promising binding modes, the 43625 poses were ranked using five different criteria:

A. *BUDE* score alone (the more negative, the better);
B. *BUDE* score, and in addition only considered if the *act*KR:Arg38:Cζ – *act*ACP:Ser42:Cβ distance falls below a 10.0 Å threshold;
C. as above, but with a 9.0 Å threshold;
D. as above, but with a 7.0 Å threshold; and
E. as above, but with a 6.0 Å threshold.

For each of the ranking criteria A-E listed above, we chose 2 to 5 models within the highest-scoring 30: very similar models by RMSD having a less favorable (i.e., more positive) *BUDE* score, as well as models already captured by one of the previous ranking criteria, are discarded. The origin of **M4-M20** is thus summarized in Table S1.

Table S1. Origin of the 17 (*act*KR–NADPH)–*act*ACP Binding Modes Chosen from Protein-protein Docking, Ordered by Model Number and *BUDE* Score.

Mode	*BUDE* *Score (kJ mol⁻¹)*	act*KR:R38:Cζ* – act*ACP:S42:Cβ Threshold*	*Ranking Criterion*	act*KR:R38:Cζ* – act*ACP:S42:Cβ Distance (Å)*	*Rank out of Best 30 in Ranking Criterion*
M5	–131.53	None	A	12.21	7[a]
M6	–125.76	None	A	16.33	9
M7	–124.55	< 10.0 Å	B	9.81	1
M8	–111.45	< 10.0 Å	B	9.91	18
M9	–103.78	< 9.0 Å	C	8.74	4
M10	–96.64	< 9.0 Å	C	7.87	7
M11	–95.88	< 9.0 Å	C	8.97	12
M12	–93.01	< 9.0 Å	C	7.44	20
M4	–88.00	< 7.0 Å	D	5.52	1
M13	–83.76	< 7.0 Å	D	6.76	4
M14	–82.51	< 7.0 Å	D	4.15	14
M15	–81.10	< 7.0 Å	D	4.01	21
M16	–80.65	< 7.0 Å	D	6.64	29
M17	–81.98	< 6.0 Å	E	5.43	6
M18	–80.99	< 6.0 Å	E	5.42	8
M19	–78.82	< 6.0 Å	E	3.69	21
M20	–78.67	< 6.0 Å	E	4.08	24

[a] *Despite the choice of a restricted number of Connolly surface points as described in the previous subsection, in all six highest-ranking models overall (BUDE score –144.35 to –132.04 kJ mol^{-1} and all structurally very similar to each other), actACP still docks in a region of actKR monomer A where it would clash with monomer B were it present.*

2.4 External Docking Models: **M1-M3**

For comparison, and to reduce bias, three extra *act*KR–*act*ACP models were added to the 17 selected from docking: two based on previously obtained substrate-free models (**M1** from ref. 7;

M2 from ref. 4), and **M3**, derived from the crystal structure of *Escherichia coli* enoyl reductase FabI, complexed to its sister ACP (PDB 2FHS).[8] Construction of **M1**-**M3** was carried out with the *Biovia Discovery Studio* package; PDB files of all three models (alongside those of our docking models **M4-M20**) are also provided as Supporting Information.

For consistency with our docking models, *act*KR–NADPH molecules in **M1** and **M2** were swapped with our reference *act*KR–NADPH structures originating from previous classical MD,[1] through alignment of NADPH heavy atoms. Similarly, for construction of **M3** our reference *act*KR–NADPH structure was overlaid on the *E. coli* FabI crystal structure, based on their respective cofactors. To reconstruct the position and orientation of *act*ACP, our reference *act*ACP model (see main text) was overlaid onto the *E. coli* FabI ACP present in 2FHS based on structural similarity, as calculated by *Discovery Studio*'s internal algorithm, which gives equal weight to sterics and electrostatics. Goodness of the overlay was assessed by visual inspection, confirming that *act*ACP helices correctly overlap with the related *E. coli* FabI ACP elements conserved in the crystal structure. After this alignment, the *act*ACP in **M3** ended up in a distal position at the tip of the α6-α7 loop: we note that, due to the larger size of the loop in *act*KR, there remains some degree of *act*ACP–*act*KR backbone overlap. This was resolved by slightly translating the *act*ACP outwards.

Finally, in all three external models, the (few) side-chain clashes between *act*ACP and *act*KR–NADPH resulting from the operations discussed above were removed by adopting the best non-clashing side chain rotamer from Ponder and Richards' rotamer library.[9]

BUDE scores (interaction energies) for **M1-M3** indicate that all three external models start at a considerable disadvantage (unfavorable [positive] scores in kJ mol^{-1}, Table S2)

Table S2. *BUDE* Scores Calculated for External (*act*KR–NADPH)–*act*ACP Binding Modes.

Mode	BUDE *Score (kJ mol^{-1})*	*Ranking Criterion*	act*KR:R38:Cζ* – act*ACP:S42:Cβ Distance (Å)*
**M1**	47.83	[None: external model]	12.26
**M2**	420.56	[None: external model]	34.01
**M3**	83.89	[None: external model]	37.74

3. Starting Structures for Classical MD: Additional Details

Construction of all starting structures was carried out using a combination of the *tleap*,[10] *CPPTRAJ*,[11] and *Biovia Discovery Studio* software. All coordinates and topologies (`.rst`, `.top`, `.pdb`) of the structures resulting from the procedures detailed in sections 3.1 and 3.2 are provided as Supporting Information.

3.1 Starting Structures for MD stages $\mathbf{I_A}$ and $\mathbf{I_B}$

Complexes of (*act*KR–NADPH)$_4$–(*act*ACP)$_4$ featured in MD runs for $\mathbf{I_A}$ and $\mathbf{I_B}$ (i.e., without PPant-octaketide) were constructed using identical procedures, the only difference being the 17 in-house or 3 external (Section 2.4 above) docking binding modes (**M1-M20**) from which they are built. (*act*KR–NADPH denotes a holo *act*KR monomer.) More specifically:

- The five (*act*KR–NADPH)$_4$–(*act*ACP)$_4$ systems in $\mathbf{I_A}$ are assembled from five quadruplets (**M4,M1-M3**; **M5-M8**; **M9-M12**; **M13-M16**; and **M17-M20**; respectively).

- The three (*act*KR–NADPH)$_4$–(*act*ACP)$_4$ systems in $\mathbf{I_B}$ are assembled from three quadruplets of **M10**, **M14**, or **M17**, respectively, with all four *act*ACP molecules in each system thus identically oriented in one of the three modes.

Each docking model (monomer) was overlaid onto one of chain A, B, C, or D in our primary reference *act*KR crystal structure (PDB 2RH4).[2] Only interfacial residues as defined for the protein-protein docking (numbered and justified in the relevant Section 2.1 above) are considered for this alignment: this successfully ensures that there is no steric clash between the reassembled (*act*KR–NADPH)$_4$ tetramers.
Due to the choice of surface points in the protein-protein docking and ranking criteria (see again Section 2.1 above), there are no steric clashes arising upon reassembly of the *BUDE* docking models. On the other hand, external model **M2**[4] ends up being sandwiched between two holo *act*KR monomers, overlapping with the newly introduced one: clashes are again resolved by moving *act*ACP slightly outwards.

3.2 Starting Structures for MD stage **II**

A. Alterations to $\mathbf{M14_{16IB}}$ tetramerization. The starting point for the construction of S_{OHax}, R_{OHax}, S_{OHeq}, and R_{OHeq} for MD simulations in **II** [i.e., (*act*KR–NADPH)$_4$–(*act*ACP)$_4$–(*XR*-$\mathbf{2}_{\mathrm{OH}yy}$)$_2$–(*XS*-$\mathbf{2}_{\mathrm{OH}yy}$)$_2$] is always the (*act*KR–NADPH)–*act*ACP model $\mathbf{M14_{16IB}}$ identified from simulations in $\mathbf{I_B}$ (see nomenclature in main text), after the clustering procedure (see Section 6). First, the α6-α7 loop and the adjacent regions (residues 188-229) are replaced by the equivalent region from an *act*KR-octaketide mimic crystal structure:[12] this is done by using backbone atoms of the two residues at its extremities (187 and 230) as a reference, overlaying them rigidly onto their counterparts in $\mathbf{M14_{16IB}}$.
Next, the following residues are altered, using appropriate sidechain rotamers from Ponder and Richards' library where applicable.[9]

- Gly201 in the *act*KR α6-α7 loop is mutated back to histidine (protonated on Nε2), adopting the rotamer most closely resembling the one in $\mathbf{M14_{16IB}}$.
- Thr145 in the active site region of the *act*KR monomer is rotated so that its hydroxyl group is approximately oriented towards the position of O11 in **2**.
- Arg72 in *act*ACP is switched to the second most populous rotamer, allowing it to make better contact with Glu200 and Asp204 side chains in the *act*KR α6-α7 loop. This is the only change in *act*ACP – *act*KR contacts arising after replacing the loop.

Once the above alterations are made to $\mathbf{M14_{16IB}}$, each identical copy is overlaid onto chains A, B, C, or D in PDB 2RH4[2] using the same procedure used to construct systems in $\mathbf{I_A}$ and $\mathbf{I_B}$.
B. Modeling 2 inside active sites. Next, using manual docking, the four active sites in the tetrameric *act*KR-*act*ACP structures generated from $\mathbf{M14_{16IB}}$ are populated with isomer-conformers of 2 as shown in Table S3.

Table S3. Starting Conformations of 2 in *act*KR for Stage II MD.

Label	***2*[a] *in actKR monomers A, B***	***2*[a] *in actKR monomers C, D***
S_{OHax}[b]	*SR*-$\mathbf{2}_{\mathrm{OHax}}$	*SS*-$\mathbf{2}_{\mathrm{OHax}}$
R_{OHax}[c]	*RR*-$\mathbf{2}_{\mathrm{OHax}}$	*RS*-$\mathbf{2}_{\mathrm{OHax}}$
S_{OHeq}[d]	*SR*-$\mathbf{2}_{\mathrm{OHeq}}$	*SS*-$\mathbf{2}_{\mathrm{OHeq}}$
R_{OHeq}[e]	*RR*-$\mathbf{2}_{\mathrm{OHeq}}$	*RS*-$\mathbf{2}_{\mathrm{OHeq}}$

[a] *Cf. Scheme 2, main text.* [b] *(actKR–NADPH)$_4$–(actACP)$_4$–(SR-$\mathbf{2}_{OHax}$)$_2$–(SS-$\mathbf{2}_{OHax}$)$_2$.* [c] *(actKR–NADPH)$_4$–(actACP)$_4$–(RR-$\mathbf{2}_{OHax}$)$_2$–(RS-$\mathbf{2}_{OHax}$)$_2$.* [d] *(actKR–NADPH)$_4$–(actACP)$_4$–(SR-$\mathbf{2}_{OHeq}$)$_2$–(SS-$\mathbf{2}_{OHeq}$)$_2$.* [e] *(actKR–NADPH)$_4$–(actACP)$_4$–(RR-$\mathbf{2}_{OHeq}$)$_2$–(RS-$\mathbf{2}_{OHeq}$)$_2$.*

Before modelling the substrate portion of **2**, we first created a "conformational template" for the PPant moiety based on crystallographic data.[12] First, $\mathbf{M14_{16IB}}$ was aligned to this structure (employing the usual reference residues, cf. Section 2.1 above). Next, we overlay the PPant arm of **2** onto the mimic (using *Biovia Discovery Studio*'s overlay tools) through heavy-atom tethers, so that rotatable bonds of **2** can be automatically rotated to match the conformation of the mimic. After this, only minor tweaks were necessary to the PPant backbone, to correct the few steric clashes arising with *act*ACP residues in $\mathbf{M14_{16IB}}$. This served as the PPant portion for all 'isomer-conformers' of **2**, with just some conformational flexibility in the border between PPant and octaketide (i.e. through dihedrals around the sulfur atom). Significant variation was present in the cyclo-octaketide portions: regardless of the stereochemistry and conformation of C7-C12 ring in **2**, it is always attempted to retain catalytic distances for ring opening and C9 ketoreduction within reasonable ranges across the active sites of all four systems. Such distances are:

- For C9 ketoreduction:
  - NADPH:H$^-$ – **2**:C9 (2.14 – 3.78 Å);
  - *act*KR:Ser144:Hγ – **2**:O9 (1.16 – 3.26 Å); and
  - *act*KR:Tyr157:Hη – **2**:O9 (1.45 – 2.97 Å).
- For C7-C12 ring ring opening:
  - *act*KR:Thr145:Hγ1 – **2**:O11 (1.33 – 3.35 Å); and
  - *act*KR:Tyr202:Oη – **2**:H7 (3.42 – 4.58 Å, accounting for a bridging water molecule).

C. Minimization. Once the four systems are constructed and solvated, a preliminary round of minimization was performed to relieve any clashes and strains resulting from "manual" docking, using several rounds of the `minimize` command in *AmberTools*' parmed utility (5 to 15 rounds, depending on the system).[10]

4. Parametrization of Isomers of 2

Five separate sets of force field parameters were produced, to be used as "building blocks": one for the PPant moiety in conjunction with *act*ACP's modified Ser42 (i.e., Ser42-PPant), and one for each of the octaketide's four possible cyclization products (*(7R,12R)*, *(7R,12S)*, *(7S,12S)*, and *(7S,12R)*). Details are provided below. All relevant libraries and parameter files of these five fragments are provided electronically.

4.1 Bonded and Lennard-Jones Parameters

All octaketide fragments, as well as the PPant portion of PPant-Ser42 (including the Oγ atom linking PPant and Ser42), were parametrized using *GAFF* forcefield parameters and atom types,[13] with standard *ff14SB* parameters[14] for the remaining Ser42 atoms (C, O, Cα, Hα, Cβ, Hβ1, Hβ2). For the one bond (Ser42:Cβ-PPant:Oγ), 3 angles, 10 torsions, and 4 impropers spanning the *ff14SB* and *GAFF* atom types, *GAFF* parameters were adopted.

4.2 Point Charges

Atomic point charges of key atoms are listed in Table S4, and compared where applicable to a previous parametrization by Maršavelski[15] of a Ser-PPant-H fragment using a similar approach, and to standard *ff14SB* parameters for unmodified Serine.[14] The following steps were used to obtain the charges:

- Fragments were taken from the modelled *act*ACP structures (i.e., modified Ser42-**2** with both adjacent residues Asp41 and Leu43, and extra fragments to neutralize charges).
    - CH_3CO-Asp41-Ser42(-*RS*-**2**)-Leu43-N(H)CH_3.
    - CH_3CO-Asp41-Ser42(-*SR*-**2**)-Leu43-N(H)CH_3.
    - CH_3CO-Asp41-Ser42(-*RR*-**2**)-Leu43-N(H)CH_3.
    - CH_3CO-Asp41-Ser42(-*SS*-**2**)-Leu43-N(H)CH_3.
- Full *Gaussian09*[16] optimizations (to confirmed minima) are carried out on the fragments at the HF/6-31G* level, with calculation of ESP charges (`Pop=MerzKollman`),[17] and information on the electrostatic potential (`IOp(6/33=2)`) collected over 10 spherical shells around each atom (`IOp(6/41=10)`), with 17 grid points per square Bohr (`IOp(6/42=17)`). All calculations are available[18] on the *ioChem-BD* server.[19]
- Resulting `.log` files were read directly by *antechamber*,[10] to assign RESP[20] charges for each fragment.
- Finally, parameter libraries produced by *antechamber* were processed as follows using *xleap*:[10]
    - Each of the four fragments is "cut" between **2**'s PPant sulfur and **2**'s octaketide C1, preserving the latter part, and discarding the former. RESP point charges for C1 and O1 were slightly adjusted: depending on their deviations from respective averages across fragments, these were made more negative to neutralize the (small) excess positive charge resulting from the CH_3CO-Asp41-Ser42-PPant-Leu43-N(H)CH_3 deletion. These changes are highlighted in Table S4.
    - RESP atomic point charges for Ser42-PPant were derived directly from the CH_3CO-Asp41-Ser42(-*SS*-**2**)-Leu43-N(H)CH_3 fragment after deletion of the CH_3CO-Asp41, Leu43-N(H)CH_3, and cyclooctaketide components. Ser42:Cβ was made somewhat more positive (in line with its *ff14SB* point charge) to neutralize the (small) excess negative charge resulting from the deletion (Table S4).

Resulting point charges are similar to either those in the standard *ff14SB* serine parametrization[14] or those in Maršavelski's parametrization[15] (cf. Table S4), save for a slightly less positive phosphorus in the latter.

Table S4. Intermediate and Final Atomic Point Charges for the Various Ser42-2 Building Blocks.[a]

Atom	*Ser ff14SB*[14]	*Ser-PPant -H*[15]	*RS-2 full*[b]	*RR-2 full*[b]	*SR-2 full*[b]	*SS-2 / S42-PPant full*[b]	*Average*[c]	*CRS*[d]	*CRR*[d]	*CSR*[d]	*CSS*[d]	*PNS*[e]
S42:N	-0.416	-0.588	-0.453	-0.386	-0.387	-0.399	-0.406	-	-	-	-	-0.399
S42:H	0.272	0.268	0.269	0.247	0.243	0.253	0.253	-	-	-	-	0.253
S42:Cα	-0.025	0.032	0.070	0.024	0.049	0.039	0.046	-	-	-	-	0.039
S42:Hα	0.084	0.096	0.125	0.126	0.119	0.124	0.124	-	-	-	-	0.124
S42:Cβ	0.212	-0.024	0.119	0.082	0.052	0.074	0.082	-	-	-	-	*0.137*
S42:Hβ1	0.035	0.075	0.070	0.087	0.095	0.088	0.085	-	-	-	-	0.088
S42:Hβ2	0.035	0.075	0.070	0.087	0.095	0.088	0.085	-	-	-	-	0.088
S42/PPant:Oγ	-0.655	-0.304	-0.585	-0.551	-0.545	-0.546	-0.557	-	-	-	-	-0.546
S42:C	0.597	0.586	0.447	0.470	0.440	0.463	0.455	-	-	-	-	0.463
S42:O	-0.568	-0.616	-0.594	-0.600	-0.596	-0.599	-0.597	-	-	-	-	-0.599
PPant:P	-	1.088	1.267	1.322	1.317	1.327	1.308	-	-	-	-	1.327
PPant:S	-	-0.383	-0.387	-0.390	-0.400	-0.309	-0.372	-	-	-	-	-0.309
Cyclo:C1	-	-	0.600	0.649	0.737	0.521	0.627	0.600	*0.572*	*0.642*	0.521	-
Cyclo:O1	-	-	-0.449	-0.518	-0.561	-0.361	-0.472	*-0.466*	-0.518	-0.561	*-0.367*	-

*[a] Numbers underlined and in italics indicate point charges that have been modified with respect to full fragment calculations, to neutralize excess charges resulting from fragment deletions. [b] RESP charges for full CH_3CO-Asp41-Ser42(-XX-**2**)-Leu43-$N(H)CH_3$ fragments. [c] Average RESP point charges across the four full fragments. [d] Final cyclo-octaketide building blocks (dangling bonds: cyclo:C1) for each of the four possible cyclization isomers. [e] Ser42-PPant building block (dangling bonds: PPant-S, S42:N, S42:C).*

5. Additional Details for MM MD Preproduction Stages ($\mathbf{I_A}$ / $\mathbf{I_B}$ and **II**)

Preproduction stages for classical MD simulations in $\mathbf{I_A}$ / $\mathbf{I_B}$ and **II**, as listed in the main text, are similar to our previous publication,[1] but with a higher target temperature (303 K instead of 298 K). In addition, simulations in **II** had the following additional restraints on key catalytic distances during most stages; force constants vary depending on the stage (see below):

- Coming into force above 2.0 Å:
  - *act*KR:Ser144:Hγ – **2**:O9;
  - *act*KR:Tyr157:Hη – **2**:O9;
  - *act*KR:Thr145:Hγ1 – **2**:O11;
  - the distance between *act*KR:Tyr202:Oη and the H1 atom of the bridging water towards **2**:H7; and
  - the distance between the O atom of the above bridging water and **2**:H7.
- Coming into force above 3.5 Å:
  - NADPH:H^- – **2**:C9.

Preproduction stages are detailed in the subsections below: unless otherwise stated, the same procedure applies to simulations in $\mathbf{I_A}$ / $\mathbf{I_B}$ and **II**.

5.1 Minimization and Solvent Equilibration

Minimization with *AMBER*'s default algorithm was performed in two stages: 1) 300 steps minimization of solvent and hydrogen atoms, with a 5 kcal mol^{-1} Å^{-2} positional restraint applied to remaining atoms; 2) 300 steps without restraints (**I**), or with the distance restraints listed above (**II**; force constant 25 kcal mol^{-1} Å^{-2}).

Solvent equilibration was performed using 9 ps of simulated annealing (*NVT* ensemble; time-step, δt, at 1 fs). Positions of all non-solvent atoms are restrained with a force constant of 10 kcal mol^{-1} Å^{-2}. Random velocities are assigned at 25 K, with the solvent heated to 400 K over 3 ps; equilibrated for another 3 ps; and finally cooled back to 25 K by the end of the 9 ps. At this stage only, temperature enforcement occurs through the Berendsen thermostat,[21] with tight temperature coupling (τ_T) for the first 6 ps (0.2 ps), and a laxer coupling (1.0 to 2.0 ps) for cooling.

5.2 Heating

For this stage, harmonic positional restraints were applied to all Cα atoms in MD simulations in both $\mathbf{I_A}$ / $\mathbf{I_B}$ and **II** (force constant 5 kcal mol^{-1} Å^{-2}); the distance restraints listed above are applied for **II** (force constant of 1.5 kcal mol^{-1} Å^{-2}). From this stage onwards, the timestep used is 2 fs, and the *SETTLE* and *SHAKE* algorithms[22] are applied to constrain all bonds containing hydrogen. Temperature is henceforth controlled *via* the Langevin thermostat (collision frequency 0.75 ps^{-1} for heating).[23] The stage entails heating systems from 25 K (upon random velocity assignment) to 303 K over 20 ps in the *NVT* ensemble.

5.3 Equilibration

Systems were equilibrated at constant pressure (*NpT* ensemble) prior to production. Pressure (1 atm) is controlled with the Berendsen barostat;[21] temperature (303 K) with the Langevin thermostat (collision frequency 1 ps^{-1}).

Equilibration runs in $\mathbf{I_A}$ / $\mathbf{I_B}$ occur over 2040ps, thus subdivided:

- 20 ps with positional restraints on Cα atoms (force constant 3.75 kcal mol^{-1} Å^{-2});
- another 20 ps with positional restraints on Cα atoms (force constant 1.75 kcal mol^{-1} Å^{-2}); and
- another 2000 ps with all restraints lifted.

Equilibration runs in **II** are 2 ns longer, thus subdivided:

- 20 ps with:
  - positional restraints on Cα atoms (force constant 3.75 kcal mol^{-1} Å^{-2}), and
  - "catalytic" distance restraints listed above (force constants of 25 kcal mol^{-1} Å^{-2});
- another 20 ps with:
  - positional restraints on Cα atoms (force constant 1.75 kcal mol^{-1} Å^{-2}); and
  - "catalytic" distance restraints still enforced with 25 kcal mol^{-1} Å^{-2};
- another 2000 ps with all positional restraints lifted, but "catalytic" distance restraints still enforced (see above); and, finally
- all restraints lifted for the final 2000 ps.

6. Additional Details for QM/MM MD Reaction Simulations (III)

6.1 Setup and Methodology

In QM/MM reaction simulations, the QM region (Figure S2) was limited to one active site in the *act*KR-*act*ACP tetramer, and comprises the cyclooctaketide moiety of **2** from C4 onwards (C4 and O15 are labeled in Figure S2); Ser144 and Tyr157 side chains from Cβ; the nicotinamide part of NADPH up to the first ribose; and four hydrogen link atoms on the QM-MM boundaries. The QM region was treated with the semiempirical method PM6[24] used and benchmarked in our previous study on *act*KR (PM6 overestimates the barrier, but the mechanism is correct).[1]

Simulations were started from 'reactive' or 'reaction competent' conformations selected from MD runs in **II**, with key interatomic distances for ketoreduction satisfied (see section 9). Simulations were run for the three isomers of **2** where reaction competent conformations are regularly sampled (see main manuscript, Figure 5). QM/MM MD umbrella sampling (US) simulations of reductive hydride transfer from NADPH to C9 in **2** were run using the difference $(x - y)$ as reaction coordinate, where y is the distance NADPH:H⁻–**2**:C9 (Figure S2; blue) and x is the distance NADPH: H⁻–NADPH:C_{H-} (Figure S2; black). The harmonic restraint on $(x - y)$ was changed in 0.1 Å steps ("windows"), starting from the value of $(x - y)$ in the chosen snapshot and sampling $(x - y)$ until 1.8 Å for 2 ps at each window, with a force constant of 100 kcal mol^{-1} $Å^{-2}$. Starting from independent reaction competent conformations, 11 or 12 US runs were collected for each chosen isomer-conformer of **2** (representing between 964 and 1062 ps of sampling along the reaction coordinate) and used for calculation of the potential of mean force (i.e., the free energy profile for ketoreduction) using the weighted histogram analysis method (WHAM),[25-26] implemented in Grossfield's code.[27] In case proton transfer between Tyr157 and O9 did not occur during US simulations, runs were discarded and replaced.

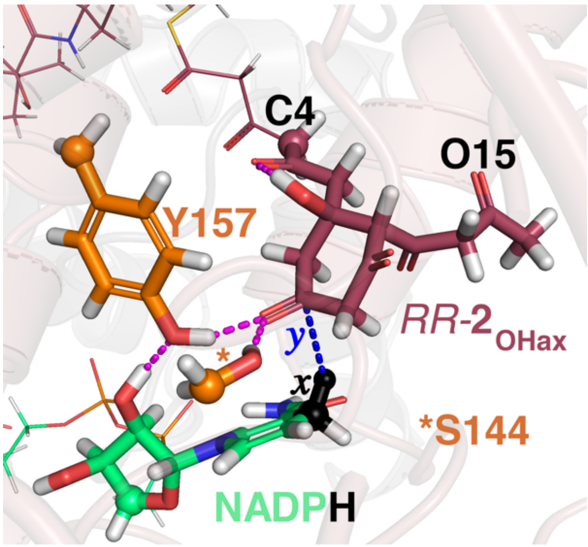

Figure S2. QM region for QM/MM MD US simulations (based on a *pro-S* reaction competent snapshot of *act*ACP-*RR*-$\mathbf{2}_{OHax}$; *i.e.*, attack from the *Re*-face of C9; see Scheme 1B and Figure 6b in the main text). QM atoms are rendered as sticks; except for NADPH: H⁻ and NADPH:C_{H-}, shown as black ball-and-stick; and four C atoms at the QM/MM boundary, rendered as spheres. Each of these four C atoms bears a link H atom to complete its valence. QM portions of (*) *act*KR:Ser144 and *act*KR:Tyr157 have C atoms in orange. All NADPH C atoms are in green (except C_{H-}); *RR*-$\mathbf{2}_{OHax}$ in dark pink. Parts of NADPH and *RR*-$\mathbf{2}_{OHax}$ falling within the MM region are rendered as lines; the remainder of the MM region has its secondary structure rendered as transparent ribbon. The chosen reaction coordinate (*x*–*y*) is marked by a black bond and a blue dotted line. Dotted magenta lines denote key hydrogen bonds. Key for non-C atoms: red: O; blue: N; white: non-hydride H; orange: P; yellow: S.

6.2 Calculated Free Energy Barriers

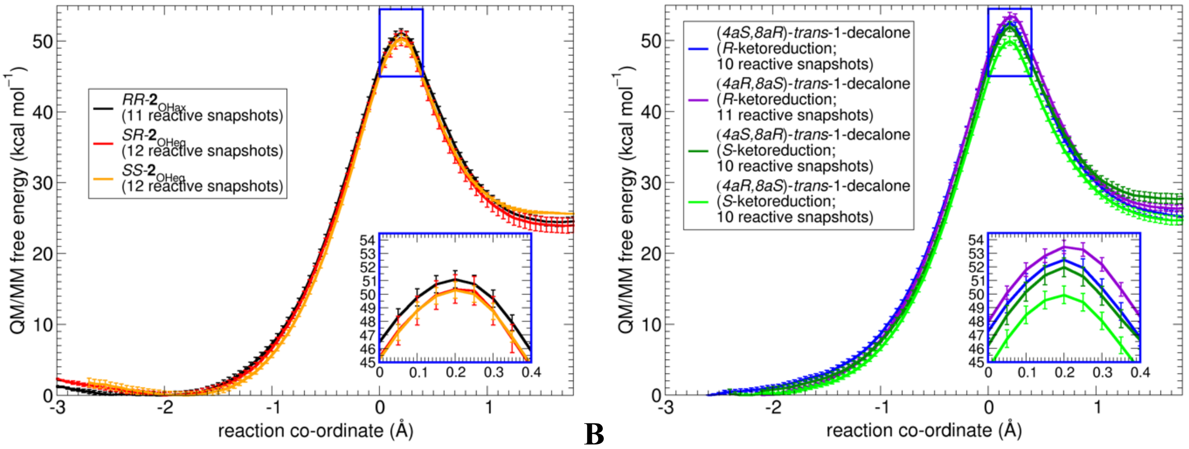

A **B**

Figure S3. A: Free energy profiles obtained from QM/MM MD US simulations in **III** (PM6/ff14SB) of the full conversion of **2** to **3**, via *S*- ketoreduction at the C9 position (main text, Scheme 1B). Each of the three curves corresponds to a different isomer-conformer of **2** (Scheme 2; black: *RR*-$\mathbf{2}_{OHax}$; red: *SR*-$\mathbf{2}_{OHeq}$; orange: *SS*-$\mathbf{2}_{OHeq}$), and represents the cumulative result of 11 or 12 valid individual runs (cf. legend and text below). Inset: zoom in on the region around the TS. Errors are determined using the Monte-Carlo bootstrap analysis (performed as described in our previous work[1]). **B:** Free energy profiles for the reduction of *trans*-1-decalone to *trans*-1-decalol (different stereoisomers), obtained for our previous work[1] using an identical level of theory. Errors are determined in an identical way to panel **A**, as described above; scales of the panel and inset are identical to panel **A**.

The 3 PMFs shown in Figure S3**A** were obtained through combined WHAM analysis of the 11 (*RR*-$\mathbf{2}_{OHax}$) or 12 (*SR*-$\mathbf{2}_{OHeq}$, *SS*-$\mathbf{2}_{OHeq}$) individual umbrella sampling (US) runs. Due to individual US runs starting from slightly different reaction coordinates, we note that some US windows below –2 may have poorer collective sampling. For comparison, Figure S3**B** shows identically obtained PMFs for the reduction of several stereoisomers of *trans*-1-decalone to *1S*- or *1R*-*trans*-1-decalol.[1]

6. Clustering of MD Runs in $\mathbf{I_A}$ and $\mathbf{I_B}$ for Evaluation of Binding Modes

The aim of MD runs in $\mathbf{I_A}$ and $\mathbf{I_B}$ was to assess the stability and validity of the 3 external and 17 *act*KR–*act*ACP binding modes (**M1-M20**) emerging from our protein-protein docking. To analyze the outcome of these simulations, the 40 $\mathbf{I_A}$ and 8 $\mathbf{I_B}$ production trajectories were clustered for each *act*KR–*act*ACP as follows:

- All 1600 frames in each 32 ns production run were aligned using the usual reference residues (listed in Section 2.1) of one of the four *act*KR–NADPH monomers present, depending on which of the 20 (*act*KR–NADPH)–*act*ACP binding modes is under study (as *per* the (*act*KR–NADPH)–*act*ACP repartition listed in Section 3.1). This amounts to 160 different alignments for the 40 $\mathbf{I_A}$ runs, and 48 different alignments for the 12 $\mathbf{I_B}$ runs (one per monomer).
- For each of these 160 + 48 alignments, clustering on the heavy atom RMSD of the *act*ACP whose binding mode is under study was performed using *CPPTRAJ*.[11] The hierarchical agglomerative clustering method was used based on the average distance between cluster members. Clustering was stopped once either 6 clusters remain or the minimum distance between each pair of clusters reached >4.0 Å.

- The cluster centroid of the largest cluster of each *act*ACP binding site simulation is used as a MD-refined binding mode, resulting in 208 different modes:
  - **M1**$_{\boldsymbol{Y}\mathbf{IA}}$-**M20**$_{\boldsymbol{Y}\mathbf{IA}}$ (***Y*** = 1-8) obtained from each of the 160 $\mathbf{I_A}$ clustering runs.
  - **M10**$_{\boldsymbol{Y}\mathbf{IB}}$, **M14**$_{\boldsymbol{Y}\mathbf{IB}}$ and **M17**$_{\boldsymbol{Y}\mathbf{IB}}$ (***Y*** = 1-16) from each of the 48 $\mathbf{I_B}$ clustering runs.

The quality of these 208 (*act*KR–NADPH)–*act*ACP MD-refined binding modes is then analyzed as discussed in the main text and the next section.

7. Evaluation of Docking Models after MD Refinement

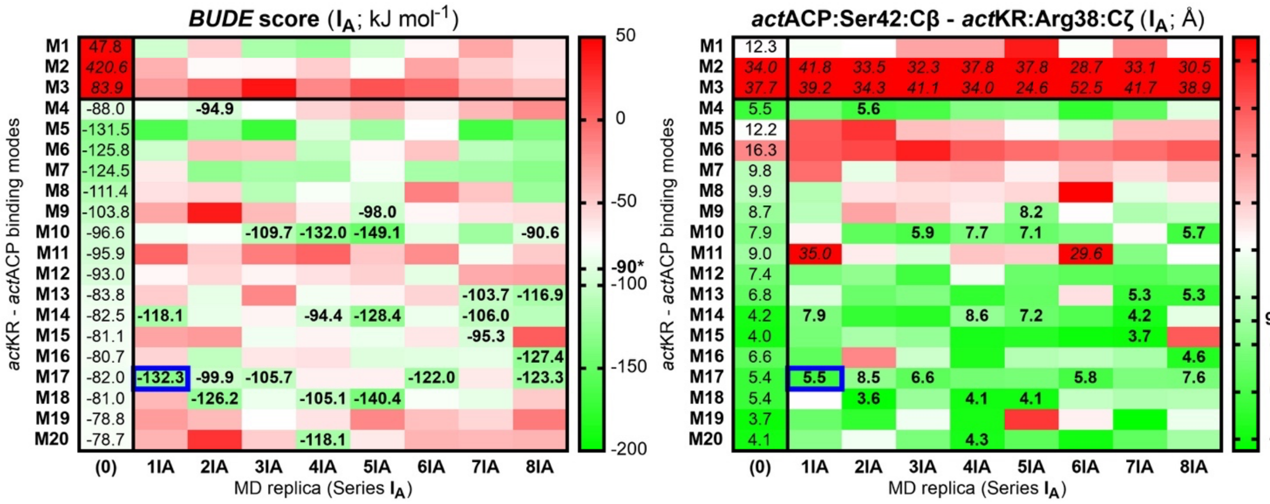

Figure S4. Evaluation of the 160 representative (*act*KR–NADPH) – *act*ACP binding modes extracted by clustering from MD simulation series $\mathbf{I_A}$ (**M1**$_{\mathbf{YIA}}$–**M20**$_{\mathbf{YIA}}$; Y = 1–8): by *BUDE* score (left; in kJ mol^{-1}) and by *act*ACP:Ser42:Cβ – *act*KR:Arg38:Cζ distance (*d*(PPant–*act*KR) or ACP-patch distance; right; in Å). Values for external modes **M1–M3** and docking modes **M4–M20** are shown in both heatmaps for comparison. The first column (**0**) indicates the values for each binding mode prior to MD. Values in **bold** in the right portion ($\mathbf{I_A}$) refer to binding modes simultaneously exhibiting a *BUDE* score below –90 kJ mol^{-1}, and an ACP-patch distance or *d*(PPant–*act*KR) of under 9 Å: both thresholds are marked with * in the color bar. Values in *italics* are off-scale (i.e., *BUDE* score of over 50 kJ mol^{-1} or *d*(PPant–*act*KR) over 21 Å). The two values framed in blue refer to **M17**$_{\mathbf{1IA}}$, selected for further validation (see Figure 4 and text in the main manuscript).

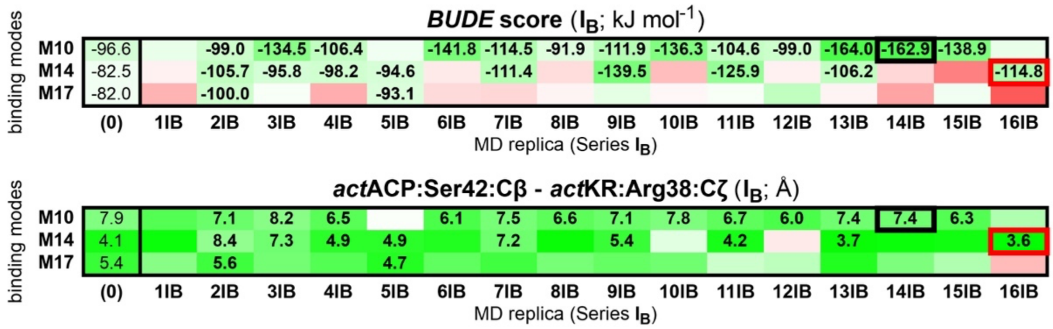

Figure S5. Ranking of the 48 additional representative (*act*KR–NADPH) – *act*ACP binding modes extracted by clustering from MD simulation series $\mathbf{I_B}$ (**M10**$_{\mathbf{YIB}}$, **M14**$_{\mathbf{YIB}}$, and **M17**$_{\mathbf{YIB}}$; **Y** = 1–16): by *BUDE* score (top; in kJ mol^{-1}); and (bottom) by *act*ACP:Ser42:Cβ – *act*KR:Arg38:Cζ distance (*d*(PPant–*act*KR); in Å). Values for each mode prior to MD are shown for comparison in the first column (**0**). Values in **bold** in the right portion ($\mathbf{I_B}$) refer to binding modes simultaneously meeting both the *BUDE* and *d*(PPant–*act*KR) thresholds described in **Figure S4** and in the main text. Color scales are identical to those in **Figure S4**; this time no value is off-scale. The framed values refer to **M10**$_{\mathbf{14IB}}$ (black) and **M14**$_{\mathbf{16IB}}$ (red), selected for further validation alongside **M17**$_{\mathbf{1IA}}$ (see Figure 4 and text in the main manuscript).

Table S5. Charged-Charged Interactions between *act*ACP and *act*KR in Selected MD-refined Models.

Model	***Residue*[a]**	***Location*[a]**	***Residue*[b]**	***Location*[b]**
$M10_{14IB}$	E47	α2 helix	K48	α2 helix
$M10_{14IB}$	R51	α2 helix	E41	α2 helix
$M10_{14IB}$	R67	α3-α4 loop	E200	α6 helix
$M14_{16IB}$	E36	(3_{10}-helix)	R93	(R patch)
$M14_{16IB}$	D41	α2 helix	R65	(R patch)
$M14_{16IB}$	R67[c]	α3 helix	NADPH[c]	–
$M14_{16IB}$	R72	α4 helix	E200	α6 helix
$M14_{16IB}$	R72	α4 helix	D204	α6 helix
$M17_{11A}$	R34	α1-α2 loop	NADPH[d]	–
$M17_{11A}$	E36	(3_{10}-helix)	R38	(R patch)
$M17_{11A}$	E36	(3_{10}-helix)	R93	(R patch)
$M17_{11A}$	D41	α2 helix	R65	(R patch)
$M17_{11A}$	R67	α3 helix	E69	α3 helix

[a] actACP. [b] actKR–NADPH. [c] Guanidinium sandwiched between the two phosphates on NADPH's adenosine ribose. [d] Guanidinium interacting with 3' phosphate on NADPH's adenosine ribose.

8. Results from NMR Titration of *act*KR and [15]N Labelled *act*ACP

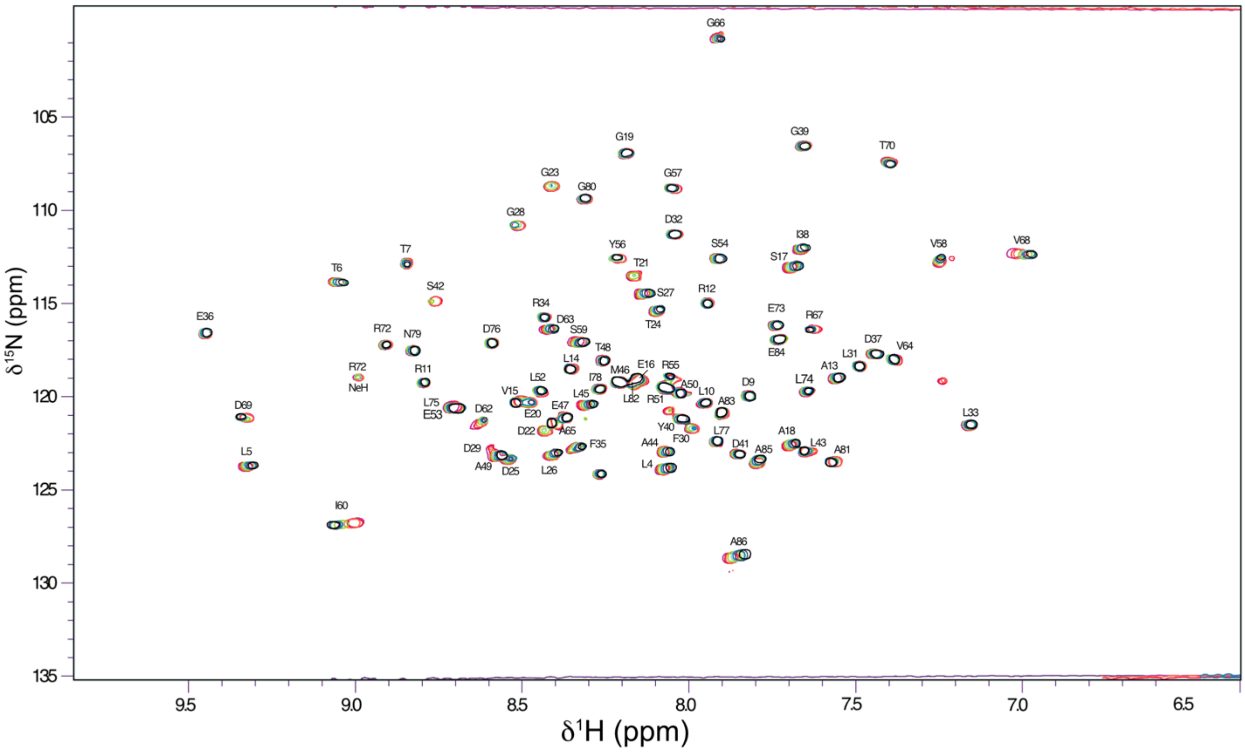

Figure S6. NMR titration experiments of actKR and ^{15}N labelled actACP. ^{1}H-^{15}N HSQC data of *act*ACP with successive additions of *act*KR. The molar ratios of *act*KR: *act*ACP at each titration point were 0.08, 0.47, 0.33, 0.67, 1.00, 1.34, 1.67 and 2.34 respectively. Concentration dependent chemical shift perturbations are indicative of a weak (e.g., mM) binding interaction.

9. Selection and Quantification of actACP-**2** Snapshots (MD stage **II**)

9.1 Definition of a Reaction Competent Pose

Any snapshot isolated from any MD simulation in **II** was considered to have the substrate in a "reaction competent" pose if the following three conditions were met simultaneously:

- the distance **2**:C9 – NADPH:H⁻ falls below 3.75 Å;
- the distance **2**:O9 – *act*KR:Tyr157:Hη falls below 3.40 Å; and
- the distance **2**:O9 – *act*KR:Ser144:Hγ falls below 2.75 Å.

As a measure of *pro-S* or *pro-R* chirality with respect to hydride attack at C9, we monitored the dihedral **2**:O9 – **2**:C10 – **2**:C9 – NADPH:H⁻, which is negative for pro-*S* (between −180° and 0°), and positive for pro-*R* (between 0° and 180°).

9.2 Quantification of Reaction Competent Poses per Isomer-Conformer

Frequencies of reaction competent poses ($\%_{reac}$) for each isomer-conformer (Table S6) were calculated cumulatively across all 8 independent MD simulations. When calculating $\%_{reac}$, it was taken into account that occasional switching between the C7-C12 ring chair conformations (*i.e.*, once or twice per simulation, or not at all) occurred, so the following pairs of isomer-conformers can interconvert:

- in systems S_{OHeq} and S_{OHax}:
  - *SR*-$\mathbf{2}_{\mathrm{OHeq}}$ ⇋ *SR*-$\mathbf{2}_{\mathrm{OHax}}$ (in active sites A and B); and
  - *SS*-$\mathbf{2}_{\mathrm{OHeq}}$ ⇋ *SS*-$\mathbf{2}_{\mathrm{OHax}}$ (in active sites C and D);
- and in systems R_{OHeq} and R_{OHax}:
  - *RR*-$\mathbf{2}_{\mathrm{OHeq}}$ ⇋ *RR*-$\mathbf{2}_{\mathrm{OHax}}$ (in active sites A and B); and
  - *RS*-$\mathbf{2}_{\mathrm{OHeq}}$ ⇋ *RS*-$\mathbf{2}_{\mathrm{OHax}}$ (in active sites C and D).

Table S6. Cumulative[a] Ring Flip Statistics vs. Frequency of (pro-*S*) Reaction Competent Poses ($\%_{\mathrm{reac}}$) for each of the 8 Isomer-Conformers of 2.

Isomer-Conformer[b]	*Relative Energy (Figure 5)*	*pro-S $\%_{reac}$ (Figure 5)[c]*	*Effective Frames[d]*	*Effective Simulation Time[d]*	*Over- or Undersampling[e]*
⇂ *RR*-$\mathbf{2}_{\mathrm{OHax}}$	0.0 kcal mol⁻¹	9.09 ± 1.56%	31155	623.1 ns	1.22
↿ *RR*-$\mathbf{2}_{\mathrm{OHeq}}$	3.4 kcal mol⁻¹	0.34± 0.06%	20045	400.9 ns	0.78
⇂ *RS*-$\mathbf{2}_{\mathrm{OHax}}$	3.2 kcal mol⁻¹	1.51± 0.42%	17199	344.0 ns	0.67
↿ *RS*-$\mathbf{2}_{\mathrm{OHeq}}$	5.5 kcal mol⁻¹	0.73± 0.17%	34001	680.0 ns	1.33
⇂ *SR*-$\mathbf{2}_{\mathrm{OHax}}$	2.5 kcal mol⁻¹	0.02± 0.01%	12598	252.0 ns	0.49
↿ *SR*-$\mathbf{2}_{\mathrm{OHeq}}$	1.4 kcal mol⁻¹	2.92± 0.86%	38602	772.0 ns	1.51
⇂ *SS*-$\mathbf{2}_{\mathrm{OHax}}$	4.3 kcal mol⁻¹	1.63± 0.63%	21100	422.0 ns	0.82
↿ *SS*-$\mathbf{2}_{\mathrm{OHeq}}$	0.9 kcal mol⁻¹	2.24± 0.58%	30100	602.0 ns	1.18

*[a] Statistics are collected across all MD simulations in Series **II**, all 8 replicas, and both active sites where an isomer of **2** can be present at any one time (i.e., sites A and B for 12R isomers; C and D for 12S; Table S3). [b] Listed by interconvertible pairs; half arrow denotes interconversion direction. [c] Calculation of $\%_{reac}$ and errors is detailed below. [d] Frames and simulation times for binding site pairs A/B and C/D are summed together. [e] Ratio compared to hypothetical absence of flips: 1 = 12800 frames in site A/C + 12800 in B/D = 25600 frames; or 256 + 256 = 512 ns.*

The eight distinct $\%_{\mathrm{reac}}$ values in Figure 5 of the main manuscript and Table S6 (one per isomer-conformer; i.e., two each from all simulations of S_{OHax}, R_{OHax}, S_{OHeq}, and R_{OHeq}) were obtained as follows:

$$\%_{reac} = \frac{N_{reac}}{N_{tot}} \times 100.$$

Here, the denominator N_{tot} denotes the total number of poses in which a particular isomer-conformer arises in any of the applicable MD replicas. Since conformers occasionally flip from axial to equatorial and vice-versa, the *observed* conformers are considered for both the numerator and denominator (rather than the initial conformer present in the starting structure of a simulation).

9.3 Errors (Leave-one-out Procedure)

Errors E in the quantification of $\%_{reac}$ for each isomer-conformer (Figure 5 and Table S6) are determined as follows, using the "leave-one-out procedure":

- for each isomer-conformer, $\%_{reac}$ is recalculated eight times as $\%_{reac-r}$ $(1 \leq r \leq 8)$ in which the rth replica is left out of the calculation.
- E on $\%_{reac}$ is given by the standard deviation of $\%_{reac-r}$ $(1 \leq r \leq 8)$.

10. QM Calculations of Isomer-Conformer Relative Free Energies

Relative free energies of the eight isomer-conformers of **2** (cf. Figure 5 and, e.g., nomenclature in Scheme 2 and Table S6) were calculated in two consecutive steps, using the *Gaussian09* package[16] and the (implicit) polarized continuum solvation model for water.[28]

- Equally sized fragments of the eight isomer-conformers (C4-C14, with capping hydrogens on C4 and C14; see inset in Figure 5) are optimized to minima—confirmed through frequency calculations—at the B3LYP/6-31+G(d,p) level of density functional theory.[29-30]
- Free energies relative to *RR*-$\mathbf{2}_{OHax}$ are estimated for all optimized fragments using entropy corrections from frequencies at the B3LYP/6-31+G(d,p) level, and single-point enthalpy calculations (potential energies and thermal corrections) at the SCS-MP2/6-31+G(d,p) level (i.e., spin-component-scaled 2nd order Møller-Plesset perturbation theory).[31-32]

Input and output files for both steps are deposited on-line[18] (*ioChem-BD*).[19] To assess energy differences as fairly as possible, H7 was always oriented towards O5 in the starting structures.

11. Analysis of Octaketide Hydrogen Bonds in MD Runs in **II**

Table S7. Residues Most Frequently[a] Donating (or Accepting) Direct Hydrogen Bonds through Octaketide Oxygens (and H7) in Isomer-Conformers of 2.

	O1	***O1***	***O3***	***O3***	***O5***	***O5***	***O7***	***O7***	***O9***	***O9***	***O11***	***O11***	***O13***	***O13***	***O15***	***O15***	***H7***[b]	***H7***[b]
	Donor	***Freq.***	***Donor***	***Freq.***	***Donor***	***Freq.***	***Donor***	***Freq.***	***Donor***	***Freq.***	***Donor***	***Freq.***	***Donor***	***Freq.***	***Donor***	***Freq.***	***Acceptor***	***Freq.***
RR-**2**$_{\mathrm{OHax}}$[c,d]	G95:H	8%	Y202:Hη	<0.5%	**2**:H7	59%	S144:Hγ	<0.5%	G146:H[e]	11%	R220:Hη[f,g]	<0.5%	F189:H	4%	R220:Hη[g]	3%	Y157:Oη	1%
RS-**2**$_{\mathrm{OHax}}$[d]	G95:H	9%	G95:H	4%	**2**:H7	27%	G146:H	<0.5%	G146:H[e]	14%	F189:H[f]	18%	**2**:H7	8%	R220:Hη[g]	7%	Y202:Oη	<0.5%
SR-**2**$_{\mathrm{OHax}}$[d]	G95:H	6%	Y202:Hη	5%	**2**:H7	6%	R220:Hη[g]	1%	F189:H[e]	10%	G146:H[f]	2%	**2**:H7	8%	R220:Hη[g]	1%	Y202:Oη	<0.5%
SS-**2**$_{\mathrm{OHax}}$[d]	G95:H	2%	**2**:H7	11%	**2**:H7	10%	Y202:Hη	3%	F189:H[e]	16%	F189:H[f]	15%	Y157:Hη	<0.5%	R220:Hη[g]	3%	Y202:Oη	<0.5%
RR-**2**$_{\mathrm{OHeq}}$[d]	G95:H	3%	Y157:Hη	2%	**2**:H7	30%	Y202:Hη	<0.5%	F189:H[e]	9%	F189:H[f]	12%	F189:H	6%	Q149:Hε	2%	Y202:Oη	<0.5%
RS-**2**$_{\mathrm{OHeq}}$[d]	G95:H	10%	**2**:H7	15%	**2**:H7	11%	Y202:Hη	<0.5%	F189:H[e]	22%	F189:H[f]	32%	T145:Hγ1	2%	R220:Hη[g]	4%	Y202:Oη	<0.5%
SR-**2**$_{\mathrm{OHeq}}$[c,d]	G95:H	7%	G95:H	9%	**2**:H7	14%	Y202:Hη	1%	F189:H[e]	9%	G146:H[f]	3%	**2**:H7	2%	R220:Hη[g]	4%	Y202:Oη	<0.5%
SS-**2**$_{\mathrm{OHeq}}$[c,d]	G95:H	5%	**2**:H7	10%	**2**:H7	25%	Y202:Hη	<0.5%	F189:H[e]	6%	F189:H[f]	12%	G146:H	<0.5%	T145:Hγ1	6%	M194:Sδ	<0.5%

*[a] To establish which contacts are the most frequent, we also assessed in how many different independent MD runs they appear: i.e., a long-lived contact appearing in just one or two individual runs was not considered to be "frequent". Average percentages for the single most frequently observed contact in each independent MD run were determined from 16 individual frequencies (2 active sites per isomer-conformer per 8 runs). [b] Only intermolecular contacts considered. [c] Isomer-conformers most frequently forming reaction competent poses for pro-S C9 ketoreduction (Figure 5 in manuscript). [d] Occasional (rare) axial-equatorial ring flips with respect to the starting conformation are included in the total count (unlike for determination of reaction competent poses). [e] Catalytically relevant hydrogen bonds with Y157:Hη and S144:Hγ, expected for ketoreduction, were also present, but not as frequently. [f] Hydrogen bonds with T145:Hγ1, possibly related to C7-C12 ring re-opening (back to **1**), were also present, and were more frequent through bridging waters (see **Table S8**). [g] Hydrogen bonds involving any of the guanidinium protons in the arginine side chain are counted.*

Table S8. Residues Most Frequently[a] Donating (or Accepting) Indirect Hydrogen Bonds, via Bridging H_2O, through Octaketide Oxygens (and H7) in Isomer-Conformers of 2.

	O1	*O1*	*O3*	*O3*	*O5*	*O5*	*O7*	*O7*	*O9*	*O9*	*O11*	*O11*	*O13*	*O13*	*O15*	*O15*	*H7*	*H7*
	Donor	***Freq.***	***Donor***	***Freq.***	***Donor***	***Freq.***	***Donor***	***Freq.***	***Donor***	***Freq.***	***Donor***	***Freq.***	***Donor***	***Freq.***	***Donor***	***Freq.***	***Acceptor***	***Freq.***
RR-**2**$_{\mathrm{OHax}}$[b,c]	G95:H	2%	Y157:Hη	2%	Y202:Hη	1%	S144:Hγ	1%	Y157:Hη	2%	T145:Hγ1	1%	R220:Hη[d]	1%	R220:Hη[d]	4%	V152:O	<0.5%
RS-**2**$_{\mathrm{OHax}}$[c]	G95:H	1%	G95:H	1%	Y157:Hη	1%	G146:H	1%	Y157:Hη	3%	T145:Hγ1	3%	R220:Hη[d]	3%	R220:Hη[d]	3%	Y202:Oη	2%
SR-**2**$_{\mathrm{OHax}}$[c]	G95:H	2%	Y202:Hη	<0.5%	V152:H	1%	R220:Hη[d]	1%	G146:H	2%	T145:Hγ1	3%	R220:Hη[d]	2%	R220:Hη[d]	2%	Y202:Oη	2%
SS-**2**$_{\mathrm{OHax}}$[c]	G95:H	1%	G95:H	1%	Y202:Hη	1%	F189:H	<0.5%	Y157:Hη	9%	T145:Hγ1	2%	G146:H	1%	R220:Hη[d]	3%	Y202:Oη	1%
RR-**2**$_{\mathrm{OHeq}}$[c]	G95:H	2%	Y157:Hη	5%	S144:Hγ	5%	Y202:Hη	<0.5%	T145:Hγ1	1%	R220:Hη[d]	3%	R220:Hη[d]	1%	R220:Hη[d]	3%	G150:O	3%
RS-**2**$_{\mathrm{OHeq}}$[c]	Y157:Hη	1%	Y226:Hη	1%	Y157:Hη	1%	Y202:Hη	<0.5%	S144:Hγ	1%	R220:Hη[d]	1%	R220:Hη[d]	2%	R220:Hη[d]	4%	Y202:Oη	4%
SR-**2**$_{\mathrm{OHeq}}$[b,c]	Y202:Hη	1%	G95:H	<0.5%	Y202:Hη	1%	Y202:Hη	<0.5%	T145:Hγ1	2%	V152:H	3%	R220:Hη[d]	3%	R220:Hη[d]	4%	Y202:Oη	3%
SS-**2**$_{\mathrm{OHeq}}$[b,c]	R34:Hη[d,e]	<0.5%	Y202:Hη	1%	Y202:Hη	2%	Y202:Hη	<0.5%	NADPH	4%	G146:H	3%	G146:H	1%	R220:Hη[d]	2%	Y202:Oη	<0.5%

[a] To establish which water bridges were the most frequent, we also assessed in how many different independent MD runs they appear: i.e., a long-lived water bridge appearing in just one or two individual runs was not considered to be "frequent". Average percentages for the single most frequently observed water bridge in each independent MD run were then determined from 16 individual frequencies (2 active sites per isomer-conformer per 8 runs). All bridges are towards actKR residues, except where noted. [b] Isomer-conformers most frequently forming reaction competent poses for pro-S C9 ketoreduction (Figure 5 in manuscript). [c] Occasional (rare) axial-equatorial ring flips with respect to the starting conformation are included in the total count (unlike for determination of reaction competent poses). [d] Water bridges involving any of the guanidinium protons in the arginine side chain were counted. [e] Water bridge with an arginine residue from actACP.

12. References

1. Serapian, S. A.; van der Kamp, M. W., Unpicking the Cause of Stereoselectivity in Actinorhodin Ketoreductase Variants with Atomistic Simulations. *ACS Catal.* **2019,** *9*, 2381-2394.
2. Korman, T. P.; Tan, Y.-H.; Wong, J.; Luo, R.; Tsai, S.-C., Inhibition Kinetics and Emodin Cocrystal Structure of a Type II Polyketide Ketoreductase. *Biochemistry* **2008,** *47*, 1837-1847.
3. Evans, S. E.; Williams, C.; Arthur, C. J.; Płoskoń, E.; Wattana-amorn, P.; Cox, R. J.; Crosby, J.; Willis, C. L.; Simpson, T. J.; Crump, M. P., Probing the Interactions of Early Polyketide Intermediates with the Actinorhodin ACP from S. Coelicolor A3(2). *J. Mol. Biol.* **2009,** *389*, 511-528.
4. Javidpour, P.; Bruegger, J.; Srithahan, S.; Korman, Tyler P.; Crump, Matthew P.; Crosby, J.; Burkart, Michael D.; Tsai, S.-C., The Determinants of Activity and Specificity in Actinorhodin Type II Polyketide Ketoreductase. *Chem. Biol.* **2013,** *20*, 1225-1234.
5. Connolly, M., Solvent-Accessible Surfaces of Proteins and Nucleic Acids. *Science* **1983,** *221*, 709-713.
6. McIntosh-Smith, S.; Price, J.; Sessions, R. B.; Ibarra, A. A., High Performance in Silico Virtual Drug Screening on Many-Core Processors. *Int. J. High Perform. Comput. Appl.* **2015,** *29*, 119-134.
7. Hadfield, A. T.; Limpkin, C.; Teartasin, W.; Simpson, T. J.; Crosby, J.; Crump, M. P., The Crystal Structure of the ActIII Actinorhodin Polyketide Reductase: Proposed Mechanism for ACP and Polyketide Binding. *Struct.* **2004,** *12*, 1865-1875.
8. Rafi, S.; Novichenok, P.; Kolappan, S.; Stratton, C. F.; Rawat, R.; Kisker, C.; Simmerling, C.; Tonge, P. J., Structure of Acyl Carrier Protein Bound to FabI, the FASII Enoyl Reductase from Escherichia Coli. *J. Biol. Chem.* **2006,** *281*, 39285-39293.
9. Ponder, J. W.; Richards, F. M., Tertiary Templates for Proteins: Use of Packing Criteria in the Enumeration of Allowed Sequences for Different Structural Classes. *J. Mol. Biol.* **1987,** *193*, 775-791.
10. Case, D. A.; Cheatham, T. E.; Darden, T.; Gohlke, H.; Luo, R.; Merz, K. M.; Onufriev, A.; Simmerling, C.; Wang, B.; Woods, R. J., The Amber Biomolecular Simulation Programs. *J. Comput. Chem.* **2005,** *26*, 1668-1688.
11. Roe, D. R.; Cheatham, T. E., PTRAJ and CPPTRAJ: Software for Processing and Analysis of Molecular Dynamics Trajectory Data. *J. Chem. Theory Comput.* **2013,** *9*, 3084-3095.
12. Zhao, S.; Ni, F.; Qiu, T.; Wolff, J. T.; Tsai, S. C.; Luo, R., Molecular Basis for Polyketide Ketoreductase-Substrate Interactions. *Int. J. Mol. Sci.* **2020,** *21*.
13. Wang, J.; Wolf, R. M.; Caldwell, J. W.; Kollman, P. A.; Case, D. A., Development and Testing of a General Amber Force Field. *J. Comput. Chem.* **2004,** *25*, 1157-1174.
14. Maier, J. A.; Martinez, C.; Kasavajhala, K.; Wickstrom, L.; Hauser, K. E.; Simmerling, C., ff14SB: Improving the Accuracy of Protein Side Chain and Backbone Parameters from ff99SB. *J. Chem. Theory Comput.* **2015,** *11*, 3696-3713.
15. Maršavelski, A., A Novel Antimicrobial Target—Expanded and Revisited Mode of Action of Pantothenamides. *RSC Adv.* **2016,** *6*, 44888-44895.
16. Frisch, M. J.; Trucks, G. W.; Schlegel, H. B.; Scuseria, G. E.; Robb, M. A.; Cheeseman, M. A.; Scalmani, G.; Barone, V.; Petersson, G. A.; Nakatsuji, H.; Li, X.; Caricato, M.; Marenich, A.; Bloino, J.; Janesko, B. G.; Gomperts, R.; Mennucci, B.; Hratchian, H. P.; Ortiz, J. V.; Izmaylov, A. F.; Sonnenberg, J. L.; Williams-Young, D.; Ding, F.; Lipparini, F.; Egidi, F.; Goings, J.; Peng, B.; Petrone, A.; Henderson, T.; Ranasinghe, D.; Zakrzewski, V. G.; Gao, J.; Rega, N.; Zheng, G.; Liang, W.; Hada, M.; Ehara, M.; Toyota, K.; Fukuda, R.; Hasegawa, J.; Ishida, M.; Nakajima, T.; Honda, Y.; Kitao, O.; Nakai, H.; Vreven, T.; Throssell, K.; Montgomery Jr., J. A.; Peralta, J. E.; Ogliaro, F.; Bearpark, M. J.; Heyd, J. J.; Brothers, E.; Kudin, K. N.; Staroverov, V. N.; Keith, T.; Kobayashi, R.; Normand, J.; Raghavachari, K.; Rendell, A.; Burant, J. C.; Iyengar, S. S.; Tomasi, J.; Cossi, M.; Millam, J. M.; Klene, M.; Adamo, C.; Cammi, R.; Ochterski, J. W.; Martin, R. L.; Morokuma, K.; Farkas, O.; Foresman, J. B.; Fox, D. J. *Gaussian 09, Revision D.01*, Wallingford CT, 2009.
17. Besler, B. H.; Merz Jr., K. M.; Kollman, P. A., Atomic Charges Derived from Semiempirical Methods. *J. Comput. Chem.* **1990,** *11*, 431-439.

18. A collection of relevant calculations is available on the io-ChemBD server. https://doi.org/10.19061/iochem-bd-6-101
19. Álvarez-Moreno, M.; de Graaf, C.; López, N.; Maseras, F.; Poblet, J. M.; Bo, C., Managing the Computational Chemistry Big Data Problem: The ioChem-BD Platform. *J. Chem. Inf. Model.* **2015,** *55*, 95-103.
20. Bayly, C. I.; Cieplak, P.; Cornell, W.; Kollman, P. A., A Well-Behaved Electrostatic Potential Based Method Using Charge Restraints for Deriving Atomic Charges: The RESP Model. *J. Phys. Chem.* **1993,** *97*, 10269-10280.
21. Berendsen, H. J. C.; Postma, J. P. M.; Van Gunsteren, W. F.; DiNola, A.; Haak, J. R., Molecular Dynamics with Coupling to an External Bath. *J. Chem. Phys.* **1984,** *81*, 3684-3690.
22. Shuichi, M.; Kollman, P. A., Settle: An Analytical Version of the Shake and Rattle Algorithm for Rigid Water Models. *J. Comput. Chem.* **1992,** *13*, 952-962.
23. Loncharich, R. J.; Brooks, B. R.; Pastor, R. W., Langevin Dynamics of Peptides: The Frictional Dependence of Isomerization Rates of N-Acetylalanyl-N′-Methylamide. *Biopolymers* **1992,** *32*, 523-535.
24. Stewart, J. J. P., Optimization of Parameters for Semiempirical Methods V: Modification of NDDO Approximations and Application to 70 Elements. *J. Mol. Model.* **2007,** *13*, 1173-1213.
25. Kumar, S.; Rosenberg, J. M.; Bouzida, D.; Swendsen, R. H.; Kollman, P. A., The Weighted Histogram Analysis Method for Free-Energy Calculations on Biomolecules. I. The Method. *J. Comput. Chem.* **1992,** *13*, 1011-1021.
26. Kumar, S.; Rosenberg, J. M.; Bouzida, D.; Swendsen, R. H.; Kollman, P. A., Multidimensional Free-Energy Calculations Using the Weighted Histogram Analysis Method. *J. Comput. Chem.* **1995,** *16*, 1339-1350.
27. Grossfield, A. *WHAM: The Weighted Histogram Analysis Method*, version 2.0.9.1; 2013.
28. Tomasi, J.; Mennucci, B.; Cammi, R., Quantum Mechanical Continuum Solvation Models. *Chem. Rev.* **2005,** *105*, 2999-3094.
29. Becke, A. D., Density-Functional Thermochemistry. III. The Role of Exact Exchange. *J. Chem. Phys.* **1993,** *98*, 5648-5652.
30. Lee, C.; Yang, W.; Parr, R. G., Development of the Colle-Salvetti Correlation-Energy Formula into a Functional of the Electron Density. *Phys. Rev. B* **1988,** *37*, 785-789.
31. Grimme, S., Improved Second-Order Møller–Plesset Perturbation Theory by Separate Scaling of Parallel- and Antiparallel-Spin Pair Correlation Energies. *J. Chem. Phys.* **2003,** *118*, 9095-9102.
32. Grimme, S.; Goerigk, L.; Fink, R. F., Spin-Component-Scaled Electron Correlation Methods. *Wiley Interdiscip. Rev.: Comput. Mol. Sci.* **2012,** *2*, 886-906.
